# Indoleacrylic acid produced by *Parabacteroides distasonis* alleviates type 2 diabetes via activation of AhR to repair intestinal barrier

**DOI:** 10.1186/s12915-023-01578-2

**Published:** 2023-04-18

**Authors:** Deliang Liu, Shaobao Zhang, Siju Li, Qian Zhang, Ying Cai, Pei Li, Hao Li, Baochun Shen, Qiongfeng Liao, Yanjun Hong, Zhiyong Xie

**Affiliations:** 1 School of Pharmaceutical Sciences (Shenzhen), Sun Yat-Sen University, Guangzhou, 510006 People’s Republic of China; 2 School of Pharmaceutical Sciences, Guangzhou University of Chinese Medicine, Guangzhou, 510006 People’s Republic of China; 3 School of Pharmacy, Kunming Medical University, Kunming, 650500 People’s Republic of China

**Keywords:** Type 2 diabetes, Intestinal barrier, *Parabacteroides distasonis*, Indoleacrylic acid, Aryl hydrocarbon receptor

## Abstract

**Background:**

Anti-inflammatory therapy is an effective strategy in the treatment of type 2 diabetes (T2D). Studies found that inflammatory responses in vivo were strongly associated with defects in the mucosal barrier function of the gut epithelium. While some microbial strains could help repair the intestinal mucosa and maintain the integrity of the intestinal barrier, the specific mechanisms remain to be fully elucidated. The present study investigated the effects of *Parabacteroides distasonis* (*P. distasonis*) on the intestinal barrier and the inflammation level in T2D rats and explored the specific mechanisms.

**Results:**

By analyzing the intestinal barrier function, the inflammatory conditions, and the gut microbiome, we found that *P. distasonis* could attenuate insulin resistance by repairing the intestinal barrier and reducing inflammation caused by the disturbed gut microbiota. We quantitatively profiled the level of tryptophan and indole derivatives (IDs) in rats and fermentation broth of the strain, demonstrating that indoleacrylic acid (IA) was the most significant factor correlated with the microbial alterations among all types of endogenous metabolites. Finally, we used molecular and cell biological techniques to determine that the metabolic benefits of *P. distasonis* were mainly attributed to its ability to promote IA generation, active the aryl hydrocarbon receptor (AhR) signaling pathway, and increase the expression level of interleukin-22 (IL-22), thus enhancing the expression of intestinal barrier-related proteins.

**Conclusions:**

Our study revealed the effects of *P. distasonis* in the treatment of T2D via intestinal barrier repairment and inflammation reduction and highlighted a host-microbial co-metabolite indoleacrylic acid that could active AhR to perform its physiological effects. Our study provided new therapeutic strategies for metabolic diseases by targeting the gut microbiota and tryptophan metabolism.

**Supplementary Information:**

The online version contains supplementary material available at 10.1186/s12915-023-01578-2.

## Background

Type 2 diabetes (T2D) is a metabolic disease characterized by chronic inflammation, insulin resistance, and islet cell damage. In susceptible individuals with T2D, the innate immune system is activated by aging, overnutrition, and other environmental factors. Then the macrophages and adipocytes secrete TNF-α, IL-6, and other inflammatory factors, which lead to insulin resistance, insulin secretion dysfunction, and metabolic syndrome [1]. Clinically, drugs with anti-inflammatory properties can reduce acute-phase reactants and glycemia significantly and possibly decrease the risk of developing T2D [2–4]. Thus, the activation of innate immune system may be an important pathogenesis of T2D, which is acknowledged by expert clinical opinion. Intestine barrier plays a key role in maintaining host-microbiota homeostasis and the reduction of inflammation in vivo. In T2D patients, gastrointestinal dyskinesia and diet cause abnormal proliferation of gram-negative bacteria, which produce a large amount of endotoxin such as lipopolysaccharide (LPS). Meanwhile, the intestinal barrier is damaged and the intestinal permeability is increased, making pathogenic factors and food antigens easier to enter the systemic circulation through the intestinal tract. It has been shown that LPS has a wide range of negative effects on human health, especially in inducing inflammation and promoting cancer initiation and progression [5, 6]. Creely et al. found that the serum lipopolysaccharide concentration was significantly higher in patients with T2D than in the control group [7]. The aforementioned results suggested that repairing the intestinal barrier to reduce inflammation in the body may be a novel idea to ameliorate type 2 diabetes.

The gut microbiota is well appreciated for its benefits on human health by promoting immune development, metabolism, and pathogen clearance [8, 9]. At present, the intervention of specific intestinal bacterial strains or enhancement of intestinal microbial ecology is a promising treatment method [10]. Numerous studies have shown that some probiotics can maintain the integrity of the intestinal mucosa and keep the permeability at normal levels. Valladares et al. gave a strain *L. johnsonii* orally to diabetic-prone rats, which were isolated from diabetes-resistant rats, and found that the expression level of intercellular tightly binding protein Claudin was increased, thus delaying or completely inhibiting the formation of diabetes [11]. Similarly, according to the research results of Moorthy et al. [12], *L. rhamnosus* and *L.acidophilus* could significantly increase the expression of tight binding proteins Claudin-1 and Occludin in rats, and play a role in repairing intestinal mucosa. Therefore, targeting intestinal flora to repair the intestinal barrier may be a new approach.

In our preliminary study [13], we investigated the effects of a traditional Chinese medicine Huang Lian Jie Du Tang (HLJDT), which consists of 4 herbs including Huanglian (*Coptidis Rhizoma*), Huangqin (*Scutellariae Radix*), Huangbai (*Phellodendri Cortex*), and Zhizi (*Gardeniae Fructus*), on type 2 diabetes in rats. We found that the abundance of *P. distasonis* was significantly negatively correlated with insulin resistance levels and blood glucose levels (Additional file 1: Figure S1) and positively correlated with tryptophan metabolites (Additional file 2: Figure S2). As one of the 18 core members in the gut microbiota of humans [14], the abundance of *P. distasonis* is also negatively correlated with obesity, nonalcoholic fatty liver, and multiple sclerosis [15–17]. Thus, we speculated that *P. distasonis* might have beneficial effects for the treatment of T2D. Previously, researchers found that *P. distasonis* could attenuate Toll-like receptor 4 signaling and Akt activation [18], and stimulate anti-inflammatory IL-10-expressing human CD4 + CD25 + T cells and IL-10 + Foxp3 + Tregs in mice [15]. Moreover, metabolomics analysis revealed that *P. distasonis* could produce succinic acid and secondary bile acids [19]. However, the direct intervention of *P. distasonis* in T2D has not been evaluated, and the relationship between *P. distasonis* and tryptophan metabolism has not been reported. Tryptophan can be metabolized to IDs by gut microbiota [20]. As the natural ligand of AhR, IDs have been reported to improve the intestine barrier by the stimulation of IL-22 production through the activation of the AhR in innate lymphoid cells (ILCs) [21–24]. Together, we further speculated that the mechanism by which *P. distasonis* improves T2D may be through the activation of AhR to repair the intestinal barrier.

In the current study, the first objective of our work was to determine whether *P. distasonis* could affect the intestinal barrier and inflammation in the high-fat diet (HFD) and streptozotocin (STZ) induced Sprague–Dawley rats. We then investigated how *P. distasonis* influenced the host from the perspective of metabolic function. Finally, we explored the specific biological role and mechanism of *P. distasonis*-related metabolites. This work illustrated how specific bacteria modulated the intestinal barrier profile. Identification of specific microbiota alterations in response to IDs may suggest new therapeutic strategies that target tryptophan metabolism for the prevention and treatment of metabolic diseases.

## Methods

### Animal experiment

Pathogen-free male Sprague–Dawley rats (body weight 130 ± 20 g) were supplied by the medical laboratory animal center of Guangdong Province (Guangzhou, China) and housed in a specific pathogen-free animal laboratory (12 h light/dark cycle, 20 °C, 50–70% humidity) with free access to food and water. All animal experimental procedures were performed at the Animal Experiment Center of Sun Yat-sen University (Guangzhou, China). The protocol was reviewed and approved by the Institutional Animal Care and Use Committee (IACUC) of Sun Yat-sen University and conformed to the National Institute of Health guidelines on the ethical use of animals. All efforts were made to ameliorate animal suffering.

**Fig. 1:**
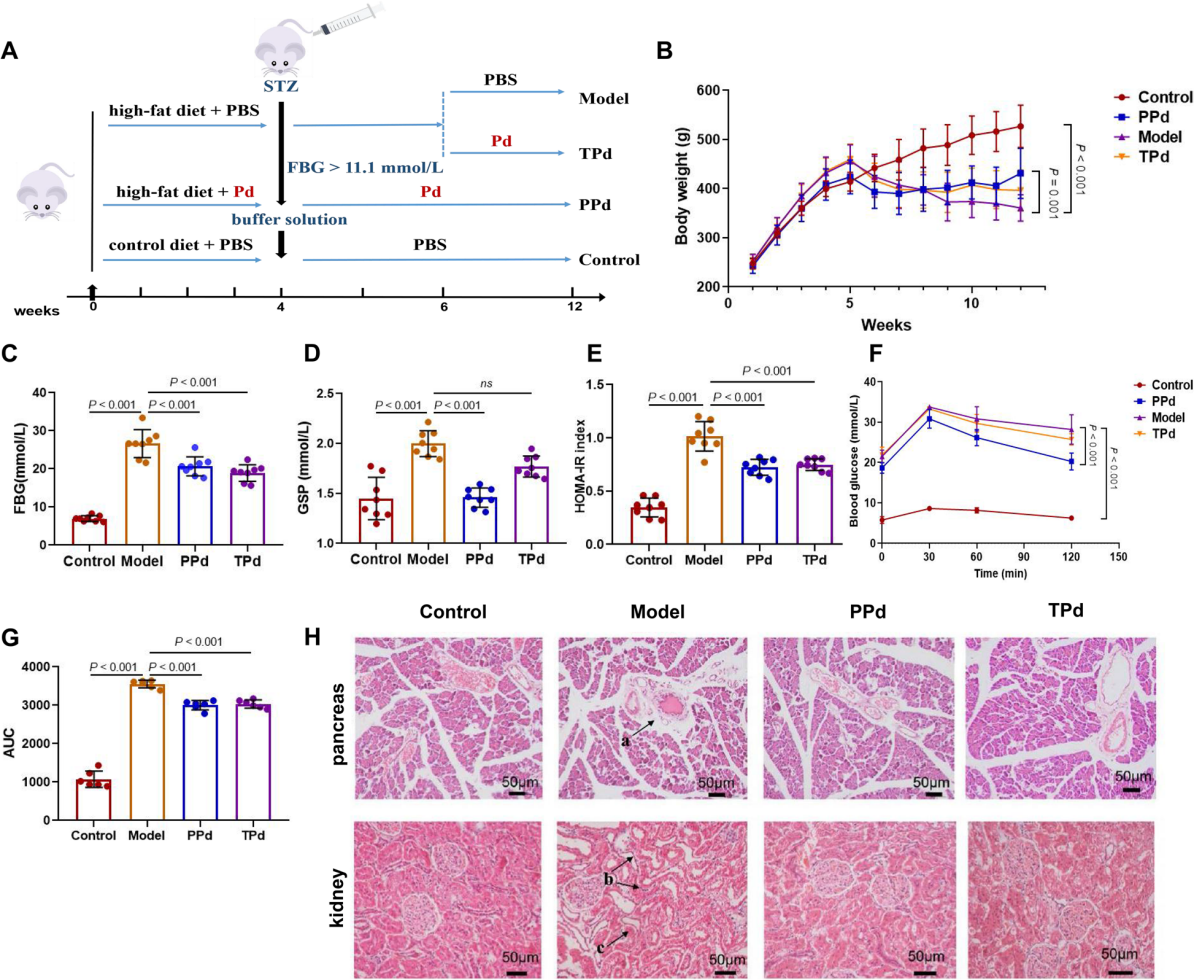
Effects of *P. distasonis* on T2D-related indicators in SD rats. **A** The overall animal experimental workflow. **B** Dynamic changes in body weight in rats (*n* = 8 per group); **C**: Fasted blood glucose in rats at week 12 (*n* = 8 per group). **D** Glycated serum protein in rats at week 12 (*n* = 8 per group). **E** Insulin resistance index in rats at week 12 (*n* = 8 per group). **F** and **G** Plasma glucose profile and mean area under the curve (AUC) measured during an oral glucose tolerance test at week 12 (*n* = 6 per group). **H** Representative images of H&E staining of the pancreas and kidney. (*n* = 6 per group), a: islet tissue, b: renal tubular epithelial cells, c: renal stroma. All differences were assessed by the Mann–Whitney *U* test. Significance was established at adjusted *P* < 0.05 with a false discovery rate (FDR) of 0.05. Data are expressed as mean ± SEM

The overall animal experimental workflow is depicted schematically in Fig. 1A. The specific procedure was as follows: After acclimatization for 7 days, a total of 32 rats were randomly divided into 3 groups. Randomization procedures Block randomization was performed through randomization.com. 8 rats were selected as the control group and given control feed. For prevention with the *P. distasonis* group (PPd group), 8 rats were fed a high-fat diet and were also given *P. distasonis* (1 × 10^9^ CFU/rat) by gavage every day until the end of the experiment. The other 16 rats were fed only a high-fat diet (HFD group). After 4 weeks, the PPd and HFD groups were fasted overnight (12 h) and received a single intraperitoneal injection of STZ (Sigma Aldrich Ltd, dissolved with 0.1 M citric acid-sodium citrate buffer (pH = 4.2–4.5) at a dosage of 45 mg/kg (bw). Rats in the control group were injected with the buffer solution only. Two weeks after the injection of STZ, HFD group rats with fasting blood glucose levels higher than 11.1 mmol/L were selected to continue the experiment. Those rats were separated into two groups (*n* = 8 each) based on their fasting blood glucose (FBG) levels and body weight. One treatment group (TPd group) was treated with *P. distasonis* via oral administration at 1 × 10^9^ CFU per rat for 6 weeks. The other treatment group (Model group) was given an equivalent volume of sterile anaerobic PBS by gavage. The control group was also given an equivalent volume of sterile anaerobic PBS by gavage. After 6 weeks of treatment, fecal samples were collected (from 8:00 am to 16:00 pm) using metabolic cages with ice-packed Eppendorf tubes and immediately stored at − 80 °C until analysis. At the end of the experiment, all of the animals were anesthetized after an overnight fast. Serum samples were collected from the orbital plexus, while pancreas, liver, and kidney tissues were harvested for further analysis.

### Oral glucose tolerance test and insulin measurement

The oral glucose tolerance test (OGTT) is not only used to diagnose diabetes or prediabetes but also used as a classical and model-based estimate of beta-cell function [24]. For the OGTT, the animals were fasted overnight (12 h) and received an oral load of 40% glucose solution (2 g/kg, Sigma-Aldrich, USA). Glucose levels were measured at four different time points in blood samples collected from the tail vein (0, 0.5, 1, and 2 h) using a glucose meter (ONETOUCH Ultra, LifeScan, USA), and the area under the curves (AUC) during the OGTT was calculated. Fasting insulin levels (FINS) were measured by ELISA kits in rats. (Nanjing Jiancheng Bioengineering Institute, Nanjing, China). The homeostatic model assessment index for insulin resistance (HOMA-IR) was evaluated using the following formula: fasting glucose (mmol/L) × fasting insulin (mIU/L)/22.5.

### Biomedical analysis

The body weight of all animals was monitored every week throughout the 12-week experiment. A glucose meter (ONETOUCH Ultra, LifeScan, USA) was used to determine the fasting blood glucose (FBG) of rats. Biochemical indexes, including total serum cholesterol (TC), triglycerides (TG), high-density lipoprotein cholesterol (HDL-C), and low-density lipoprotein cholesterol (LDL-C), were measured according to standard routine procedures on a Beckman CX5 automatic biochemical analyser (Beckman Coulter, Inc., USA). Serum cytokines were detected by the suspension chip method (Bio-Plex 200).

### Histopathological assessment

HE staining was conducted according to routine protocols. Briefly, after deparaffinization and rehydration, the kidney and pancreas sections were stained with hematoxylin solution (ZSGB-BIO, China) for 5 min followed by 5 dips in 1% acid ethanol (1% HCl in 75% ethanol) and then rinsed in distilled water. Then the sections were stained with eosin solution (ZSGB-BIO, China) for 3 min and followed by dehydration with graded alcohol and washing with xylene. The mounted slides were then examined and photographed using LEICA DM3000 LED. Evaluation was carried out by the Animal Experimental Center of Sun Yat-sen University.

### Microbial strains

*Pararabacteroides distasonis* (ATCC 8503) was purchased from ATCC (Manassas, VA), routinely cultured on blood agar plates (5% sheep blood) and incubated in an anaerobic containment system at 37 °C. *P. distasonis* stocks were prepared by freezing aliquots from the plate at − 80 °C in 50% glycerol/LB. The storage tube was taken from the − 80 °C refrigerator and quickly placed in a 37 °C water bath to prepare the bacterial solution. After melting completely, cultures were harvested at 10,000 × g for 10 min at 4 °C and washed twice in 10 mM sterile ice-cold PBS at pH 7.5.

### Cell culture studies

The colorectal adenocarcinoma cell line Caco-2 (ATCC, Manassas, VA) was cultured in DMEM (Corning, NY) supplemented with 10% fetal bovine serum (Gibco/Life Technologies, Grand Island, NY), 2 mmol/L L-glutamine (Gibco/Life Technologies, Grand Island, NY), and 1% penicillin–streptomycin (Gibco/Life Technologies, Grand Island, NY). Cell lines were monitored routinely and were free of Mycoplasma infection. All cells were maintained at 37 °C with 5% CO_2_ and were used between passages 3 and 20.

To study the intestinal permeability, Caco-2 cells were allowed to attach overnight. Then, the cells were pretreated with E. coli LPS (100 µg/ml, variant O11:B4, Sigma Aldrich, St. Louis, MO, USA) or vehicle for 24 h and then challenged with vehicle (media) or aryl hydroxyl receptor antagonist (10 µM) for 2 h. Finally, cells were treated with vehicle (media) or IA (50 µM) for 24 h.

### RNA extraction and quantitative real-time PCR (qPCR) analysis

Colon RNA was extracted according to previous literature [25]. The RNA concentration was determined using a NanoDrop ND-1000 (NanoDrop Technologies), and reverse transcription was performed with a reverse transcriptase kit (Toyobo, Osaka, Japan) according to the manufacturer’s instructions. Real-time PCR was performed with a SyBr Green PCR system (SYBR® Green Real-time PCR Master Mix; Toyobo, Osaka, Japan). The primer sequences are described in the supplementary material (Additional file 3 and Additional file 4: Table S1 and S2). GAPDH was amplified as an internal reference, and the relative expression of mRNA to GAPDH was calculated by the 2^−△△Ct^ method. Three technical replicates were performed for all the qPCR experiment and three parallel holes were set for each sample.

### Western blot (WB) analysis

Colon proteins were extracted according to tissue protein extraction protocols. Caco-2 cells were plated in six-well tissue culture plates and lysed using 200 µL radioimmunoprecipitation assay buffer (RIPA) containing protease inhibitors (Thermo Scientific, Rockford, IL, USA). Protein concentrations were determined using the Protein Assay Kit and adjusted to a known concentration before electrophoresis. WB analysis was conducted based on the procedures reported by Chen [26] with slight adjustment, and β-actin antibody as the internal reference for equality of sample loading. All primary antibodies were incubated overnight at 4 °C in antibody diluent and then incubated with the secondary antibody for 2 h at room temperature. The chemiluminescence of immunoreactive protein bands was captured with a chemiluminescence imager, and signal intensities were quantified using ImageJ 1.80 software. The protein expression was normalized to β-actin. The information of antibodies was shown in additional file 5: Table S3. Three technical replicates were performed for all the western blot experiment.

### Immunohistochemistry and immunofluorescence analysis

For immunohistochemistry, colon tissue sections were de-waxed and rehydrated before antigen retrieval with sodium citrate buffer (pH = 6.0). Sections were treated with 0.3% hydrogen peroxide in methanol for 15 min to inactivate endogenous peroxidases. They were then blocked with 1% bovine serum albumin (BSA) at room temperature for 1 h, and then incubated at 4 °C overnight with primary antibodies diluted with 1% BSA. On the following day, sections were incubated with secondary antibody diluted in 1% BSA at room temperature for 1 h. All slides were washed 3 (5 min per wash) times with Tris-buffered saline with 0.5% Tween-20 (TBST) after incubation with H_2_O_2_, primary and secondary antibodies. Finally, the sections were stained with a DAB kit (Invitrogen, CA, USA) under a light microscope at room temperature for 3 min, and the reaction was quenched with running tap water. Figure S3 showed the average optical density (AOD) (integrated optical density/area) of the ZO-1 and Occludin in colon were calculated using Image J software (Image J 1.8.0, NIH, USA).

For immunofluorescence: Cell climbing slices were washed with 0.3% Triton X-100 in PBS and blocked in 5% normal goat serum and 0.3% Triton X-100 in PBS for 2 h at room temperature. The cell climbing slices were then incubated with primary antibody in the blocking buffer overnight at 4 °C. Slices were washed in PBS and incubated with secondary antibody diluted in blocking buffer at room temperature for 2 h. Finally, DAPI (Beyotime, Shanghai, China) was used to label the nuclei at room temperature for 30 min.

### Sample preparation for HPLC–MS analysis

Serum sample preparation: 100 µL of serum samples was diluted fivefold with methanol. After adding 10 µL of the internal standard mixture, the samples were vortexed for 5 min, homogenized at − 20 °C for 30 min, and centrifuged at 13,000 rpm for 10 min at 4 °C. The supernatant was transferred out for centrifugation at 13,000 rpm for 10 min again and eventually transferred to a glass HPLC vial.

Feces samples were freeze-dried. 100 mg of fecal sample was spiked with 10 μL of internal standard solution and 590 μL of methanol–water (1:1, v/v). The mixture was vortexed for 5 min, homogenized at − 20 °C for 30 min, and centrifuged at 13,000 rpm for 10 min at 4 °C. Next, the supernatant was transferred to a new tube and centrifuged at 13,000 rpm for 10 min at 4 °C. Two microliters of supernatant were injected for LC–MS/MS analysis.

Bacterial culture sample preparation: Single colonies grown on blood agar plates were transferred to 15 mL of brain heart infusion broth, and cultures were grown on a shaker for 48 h in an anaerobic environment at 37 °C. One hundred microliters of the supernatant was transferred out and mixed with 500 μl methanol. After vortexing, the mixture was centrifuged for 10 min at 13,000 rpm at 4 °C. Then, the supernatant was transferred and passed through a 0.22-µm filter. Ten microliters of supernatant were injected for LC–MS/MS analysis.

### LC–MS/MS analysis

Sample analysis was performed on Shimadzu UPLC-MS/MS system (Kyoto, Japan) consisting of two LC-30AD pumps, a DGU-20A degasser, a SIL-20AC autosampler, a CTO-20AC column oven, a CBM-20 controller and an MS-8060 mass spectrometer. Instrument control, data acquisition, and data analysis were performed with LabSolutions software.

A Kinetex Phenyl-Hexyl column (150 × 2.1 mm, 1.7 μm, Phenomenex) was applied for chromatographic separation. Aqueous 2 mM ammonium formate with pH 5.2 (Solvent A) and methanol (solvent B) were used as mobile phases with a flow rate of 0.2 mL/min under gradient elution: 0–2.5 min, 50% B; 2.5–2.6 min, 50% B → 80% B; 2.6–5 min, 80% B; 5–5.1 min 80% B → 50% B; 5.1–8 min 50% B. The column temperature was 30 °C, and the injected sample volume was set at 2 μL. The sample vials were maintained at 4 °C in a thermostatic autosampler. The MS data was monitored using multiple reaction monitoring (MRM) mode. The optimal MS parameters were as follows: capillary voltage, 3.0 kV; nebulizing gas flow rate, 3.0 L/min; heating gas flow rate, 10.0 L/min; drying gas flow rate, 10.0 L/min; source temperature, 300 °C; heat block temperature, 400 °C. The MRM parameters for the precursor ions and product ions of all analytes and IS are shown in Additional file 6: Table S4.

### 16 s rRNA gene sequencing

Raw reads were processed with an in-house bioinformatics pipeline as previously described [27]. First, raw data were identified according to the barcode tagged with sample information, and the dates were merged to tags using Fast Length Adjustment of Short Reads (V1.2.7,http://ccb.jhu.edu/software/FLASH/) [28]. Then, chimeric sequences were discarded by the UCHIME algorithm [29]. The remaining reads were classified into operational taxonomic units (OTUs) with 97% sequence similarity by applying an open-reference OTU picking strategy with QIIME v2.0 [30] and then classified to different levels by comparison to the GreenGenes database using PyNAST software (V1.2). FastTree was used to generate the phylogenetic tree prior to the diversity analysis. Alpha diversity analysis and beta diversity analysis were calculated by QIIME software and visualized by R software (Version 3.4.1). Potential microbial biomarkers between different groups were identified through linear discriminant analysis effect size (LEfSe) with an effect size threshold of 3 [31].

## Results

### *P. distasonis* alleviated symptoms in T2D rats induced by HFD-STZ

Animal experiments were performed after 1 week of adaptive feeding, 8 rats were selected as the control group and the remaining 24 rats were used to establish the T2DM model for 6 weeks and randomly divided into the model, prevent group, and treatment groups. The prevent group was treated with *P. distasonis* before modeling, while the treatment group was treated with *P. distasonis* after modeling. At the 6th week of the experiment, compared with the control group, the model rats showed a significant decrease in body weight and significantly higher FBG. At the same time, wet cage padding was observed, indicating a significant increase in urine output. The above indicators were typical symptoms of T2D, indicating that the model of T2D rats in this study was successfully established.

To evaluate the effects of *P. distasonis* on T2D, high-fat diet, and STZ-induced rats were given vehicle (PBS) or *P. distasonis* by daily oral gavage. Compared to the vehicle-treated group (Model group), the prevention group (PPd group) showed a significant increase in weight gain (Fig. 1B). While administration of *P. distasonis* could prevent weight loss due to type 2 diabetes with different degrees, there was no statistically significant difference between PPd and TPd groups. Although the intervention time of PPd and TPd groups was different, *P. distasonis* may have a strong ability to regulate energy intake, so there is no difference in the effects of weight management between the two groups, which can also be explained by the better effects of the two groups on lipid metabolism in Table S5. Blood glucose control was monitored by fasted blood glucose (FBG), glycated serum protein (GSP), insulin resistance index, and oral glucose tolerance test (OGTT). GSP is an important indicator to assess short-term abnormal glucose metabolism [32]. The OGTT is a glucose load test, which is currently recognized as the gold standard for the diagnosis of diabetes. As shown in Fig. 1C–G, the levels of FBG, GSP, and the insulin resistance index were significantly reduced in the PPd group. Meanwhile, area under the curve of OGTT showed that glucose intolerance was significantly improved in the PPd group. Similarly, TPd group showed a similar effect in blood glucose control except for GSP.

T2D is categorized as a disorder of energy metabolism, not only on glucose metabolism, but also on lipid metabolism. The level of blood lipid can be used as a reference index of various diseases to evaluate the level of lipid metabolism. Therefore, total cholesterol (TG), triglyceride (TC), low-density lipoprotein cholesterol (LDL-C), and high-density lipoprotein cholesterol (HDL-C) were measured in this study, as shown in Additional file 7: Table S5. Compared with the control group, TG, TC, and LDL-C levels in the T2D model group were significantly increased while HDL-C showed a down-regulate trend with no statistical significance. With the *P. distasonis* intervention, the TG, TC, and LDL-C levels in the PPd and TPd groups were significantly decreased, indicating that *P. distasonis* could ameliorate lipid-metabolism disorders in T2D rats.

People with T2D often experience a series of complications due to long-term high blood glucose levels, resulting in damage to the pancreas, kidney, and other organs [33–35]. Therefore, a histopathological examination was performed to evaluate the effect of *P. distasonis* on tissue damage in T2D rats. The tissue architecture of the pancreas and kidney from untreated or *P. distasonis*-treated rats was evaluated to assess whether *P. distasonis* treatment could ameliorate tissue damage. As shown in Fig. 1H, the tissue architecture in the pancreas and kidney were protected in the PPd and TPd groups. No fibrosis and necrotizing changes were observed in the pancreatic islets of rats in the control group, no obvious vacuolar degeneration of acinar cells, and no collagen hyperplasia was observed in the interstitium of the islets. In the model group, the pancreatic acinar cells showed vacuolar degeneration (20–30%), the islet interstitial fat deposition, and the islet structure fibrosis or even disappearance. In the PPd group, some acinar cells showed vacuolar degeneration (5–10%), and no collagen fiber hyperplasia was observed in the islet interstitium. In the TPd group, vacuolar degeneration of some acinar cells (10–20%), collagen hyperplasia, and islet fibrosis were observed in the islet interstitium. For the kidney, there were no obvious pathological changes in renal tubular epithelial cells in the renal cortex, no dilatation of renal tubules, and no proliferation of interstitial fibers in the control group; In the model group, necrosis of renal tubular epithelial cells (30–40%), dilatation of some renal tubules (20–30%), vacuolar degeneration of epithelial cells (30–40%), and interstitial fibrous tissue hyperplasia (20–30%) were observed; In PPd group, renal tubular epithelial cell necrosis (5–10%), vacuolar degeneration of some epithelial cells (20–30%), renal tubular dilatation and interstitial fibrous tissue proliferation were not observed; Renal tubular epithelial cell necrosis (10–20%), vacuolar degeneration of some epithelial cells (20–30%), renal tubular dilatation (5–10%), and interstitial fibrous tissue hyperplasia (5–10%) were observed in TPd group. Histological lesions were less severe in all treated groups than the lesions in the model group. Meanwhile, Table S5 showed that the levels of serum albumin (ALB) and blood urea nitrogen (BUN) in T2D rats were greatly increased compared with those in the control and PPd groups, which also demonstrated the protective effect of *P. distasonis* on the kidney. Taken together, *P. distasonis* treatment effectively reversed the features of T2D in SD rats.

### *P. distasonis* regulated intestinal flora disorder in T2D rats

At the end of the animal experiment (12 weeks), we collected feces from four groups of rats. DNA extraction, PCR amplification, and 16 s sequencing were performed by Huada Genomics Co., Ltd (Shenzhen China). After sequencing, a total of 1,625,456 raw data points were obtained from 24 fecal samples in the 4 groups, with an average sequence length of 2982 bp. A total of 892,419 high-quality sequences were obtained after filtering and removing chimeras by DADA2. Bioinformatics analysis was conducted to obtain diversity analysis results.

**Fig. 2:**
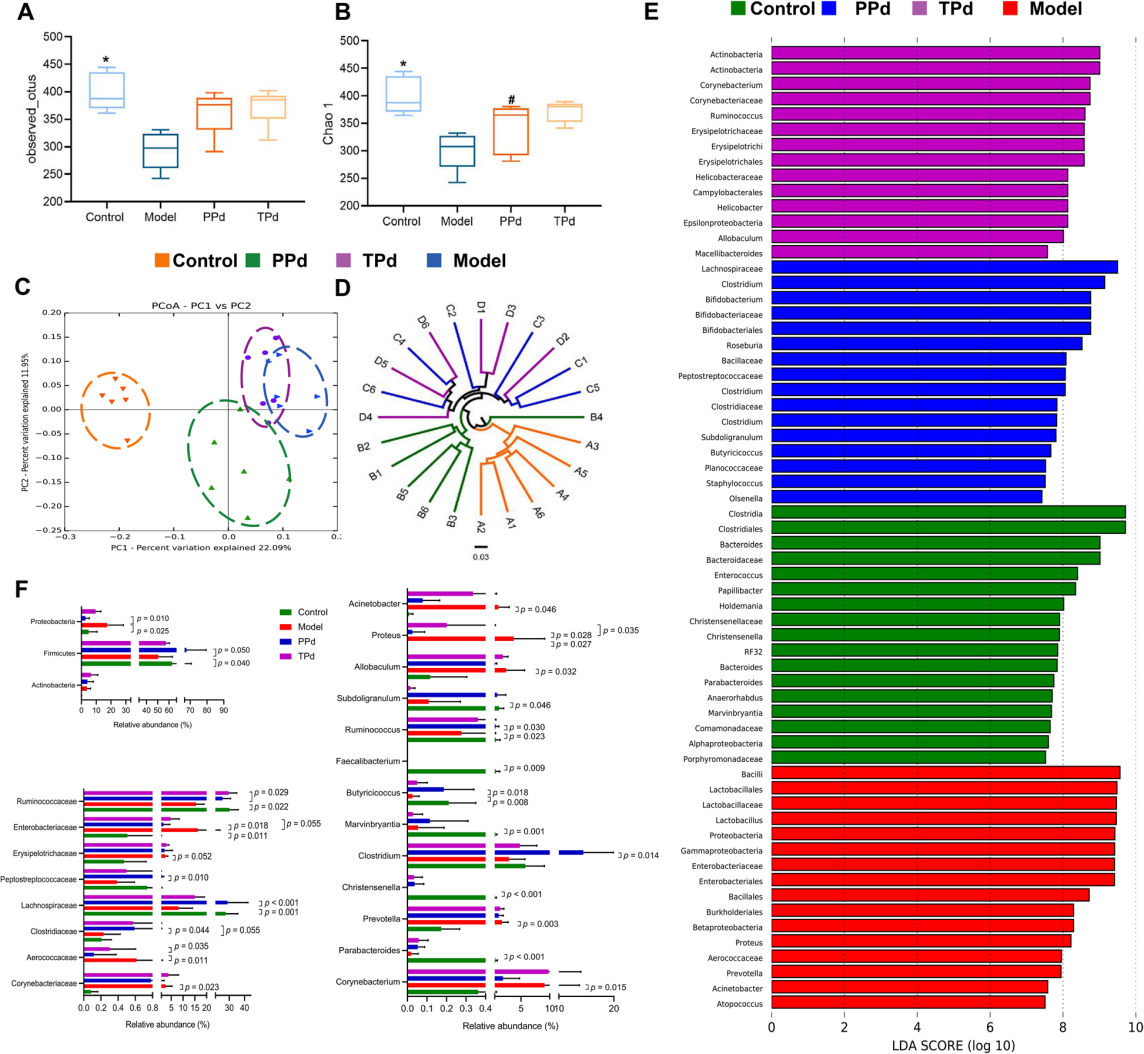
Structural comparison of fecal microbiota among the four groups. **A** and **B** Observed OTUs and Chao 1 index; Statistical analysis was performed using Tukey–Kramer post hoc test. Values are means ± SD (*n* = 6). **C** Principle coordinate analysis (PCoA) plot generated using OTU metrics based on the Bray–Curtis similarity for control, model, and PPd and TPd groups (*n* = 6 per group). **D** Hierarchical clustering based on the Bray–Curtis similarity of fecal microbial composition among all groups (*n* = 6 per group). **E** Linear discriminant analysis (LDA) effect size method was performed to compare taxa between control, model, and PPd and TPd group (*n* = 6 per group). The bar plot lists the significantly differential taxa based on effect size (LDA score (log 10) > 3). **F** Comparison of relative abundance at phylum, family, and genus levels among all groups (*n* = 6 per group). Data are expressed as mean ± SEM. Differences were assessed by the Mann–Whitney *U* test. Significance was established at adjusted *P* < 0.05 with a false discovery rate (FDR) of 0.05

As shown in Fig. 2A, B, the OTUs and Chao1 index were significantly different between the model group and the control group. The results demonstrated that the species richness and diversity were significantly decreased after modeling. However, higher levels of OTUs and Chao1 index were observed in the PPd group and TPd group, but not statistically significant compared with the model group, indicating that *P. distasonis* intervention could not significantly improve the species richness and diversity in T2D rats.

To analyze the overall structure of the gut microbial community, beta diversity was evaluated with PCoA based on UniFrac distance (Fig. 2C). As shown in the PCoA plot, the control group was clearly separated from the other groups. Compared with the model group, the PPd group was closer to the control group, indicating that the species structure between the two groups was similar. Compared with the PPd group, the microbial community structure in the TPd group was similar to that in the model group, suggesting that the longer the intervention cycle of *P. distasonis*, the greater influence on the intestinal flora. The hierarchical clustering graph further verifies the results of PCoA (Fig. 2D). Our data demonstrated that *P. distasonis* could improve the intestinal microflora structure of T2D rats, and its preventive effect was better than its therapeutic effect in regulating the intestinal microflora.

To further investigate the effect of *P. distasonis* on the intestinal flora in T2D rats, we carried out a statistical analysis using LEfSe analysis. The difference analysis was conducted on the bacterial community among groups to find the species characteristics that could best explain the difference among groups according to Linear Discriminant Analysis (LDA) score (LDA > 3). A total of 63 key phylotypes with significant differences were screened at different levels, including 17 in the normal group, 16 in the model group, 16 in the PPd group and 14 in the TPd group (Fig. 2E). GraphPad Prism software was used to visualize the relative abundance of bacteria with significant differences in LEfSe analysis at the phylum, family, and genus levels (Fig. 2F). At the genus level, compared with the control group, the relative abundance of *Prevotella*, *Acinetobacter*, *Allobaculum*, *Corynebacterium*, and *Proteus* was significantly decreased in the model group. Meanwhile, the relative abundance of 6 genera in the model group was decreased significantly compared with the control group, including *Subdoligranulum*, *Ruminococcus*, *Marvinbryantia*, *Christensenella*, and *Faecalibacterium*. With the treatment of *P. distasonis*, the above-mentioned bacteria in the PPd and TPd groups tended to a normal level, which exhibited a better effect in the PPd groups than that in the TPd group.

### *P. distasonis* improved intestinal tight junction function in T2D rats

**Fig. 3:**
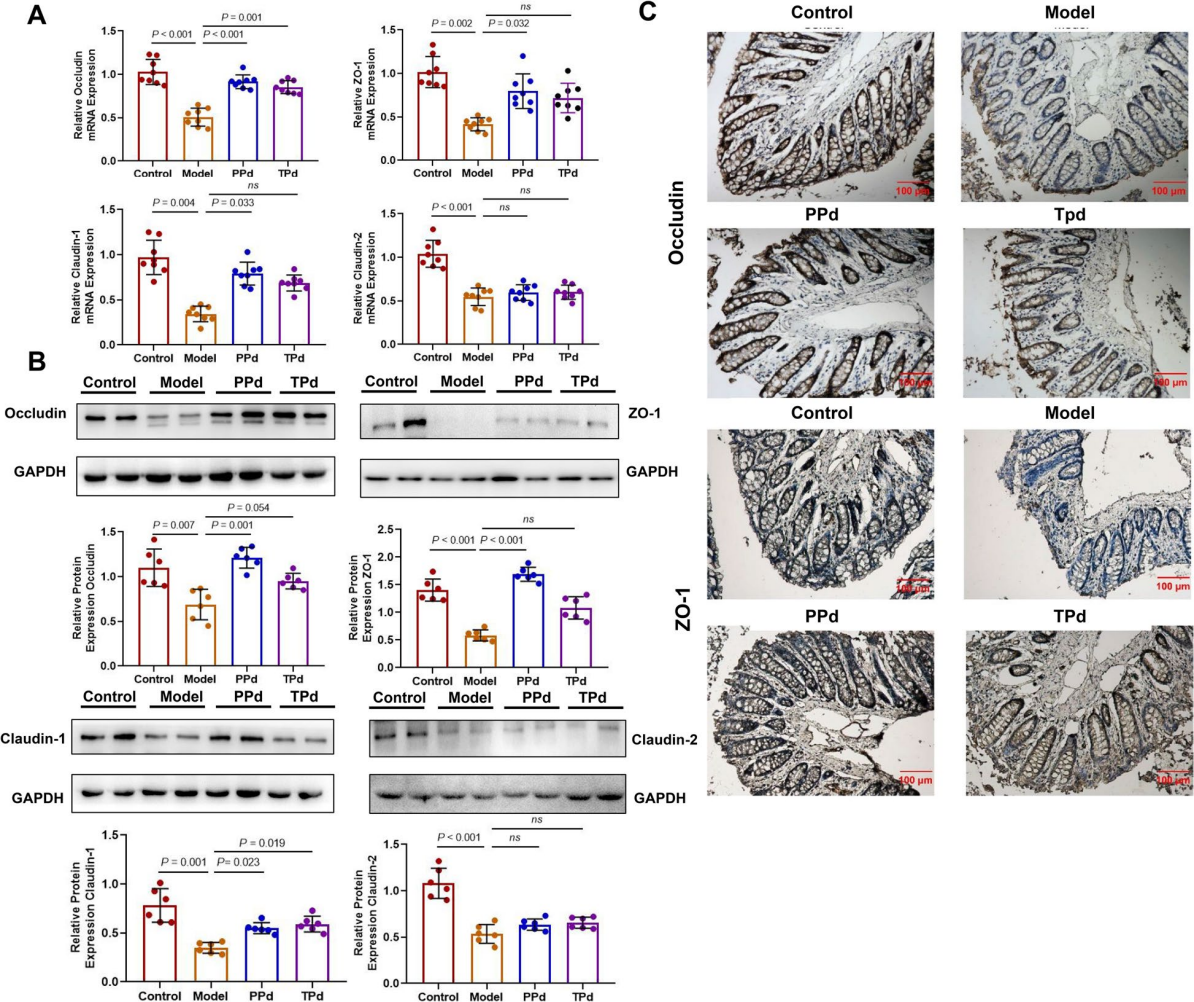
Protective effects of *P. distasonis* against T2D on colon barrier disruption in rats. **A** Relative mRNA expression of Occludin, Claudin-1, Claudin-2, and ZO-1 proteins in colon tissue of rats (*n* = 8 per group). Each dot shows the value from each independent replicate. **B** Representative Western blot and densitometric analyses of Occludin, Claudin-1, Claudin-2, and ZO-1 proteins in colon tissue of rats (*n* = 6 per group). Each dot shows the value from each independent replicate. **C** Representative pictures of immunohistochemical staining for Claudin-1 and ZO-1 on colon tissue (n = 6 per group). (After DAB staining, the positive antibody reaction was brown. After restaining with hematoxylin, the nuclei were blue). Data are expressed as mean ± SEM. Differences were assessed by the Mann–Whitney *U* test. Significance was established at adjusted *P* < 0.05 with a false discovery rate (FDR) of 0.05

To investigate the relationship between T2D and intestinal barrier function in vivo, the relative expression levels of occludin, claudin-2, claudin-1, and ZO-1 protein in colonic tissue of rats at week 12 were examined by qPCR and WB analysis. As shown in Fig. 3A, compared with the control group, the relative expression of Occludin, Claudin-1, Claudin-2, and ZO-1 mRNA was significantly downregulated in the model group at the transcriptional level while different administration schedules of *P. distasonis* upregulated the above relative expression except for Claudin-2. The WB analysis was consistent with those of qPCR (Fig. 3B). Compared with the PPd group, a similar trend was noticed in the TPd group. At the transcriptional level, the expression level of Claudin-1, Occludin, and ZO-1 was increased in the TPd group, but no statistical significance was observed for Claudin-1 and ZO-1. However, at the protein level, only Claudin-1 was significantly increased. Immunohistochemical results of the colon tissue showed that Occludin and ZO-1 were mainly expressed in the membrane and space of the colon epithelium (Fig. 3C). As described in Fig. 3C, T2D rats treated with *P. distasonis* showed great improvement in intestinal barrier function, as demonstrated by high expression of tight junction (TJ) proteins (Occludin and ZO-1). As shown in Additional file 8: Figure S3, there was a significant difference in the AOD of the Occludin and ZO-1 among the four groups. The AOD of the Occludin and ZO-1 expression was significantly lower in the model group, compared with the other three groups.

Taken together, at the protein and transcriptional levels, the improvement of intestinal barrier function in the TPd group was less significant than that in the PPd group. Immunohistochemical results further showed that the expression of colonic tight junction proteins was increased in both the PPd and TPd groups, suggesting that *P. distasonis* could improve the tight junctions of the intestinal barrier.

### *P. distasonis* reduced inflammation and endotoxin levels in T2D rats

**Fig. 4:**
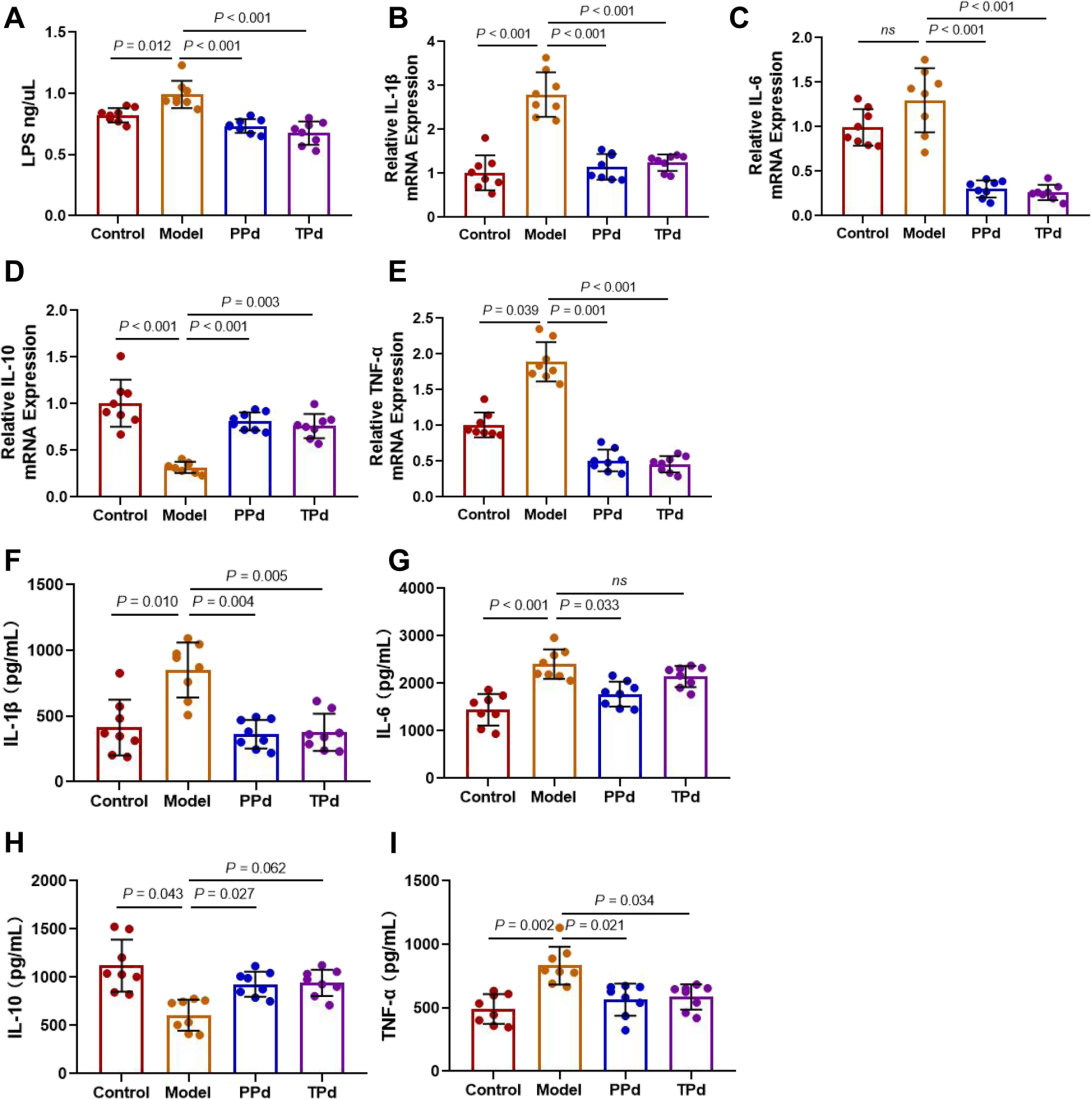
The regulation of *P. distasonis* on inflammatory factors in type-2 diabetes rats. **A** The level of LPS in plasma (*n* = 8 per group). Each dot shows the value from each independent replicate; **B**–**E** Relative mRNA expression of IL-1β, IL-6, IL-10, and TNF-α in colon tissue of rats (*n* = 8 per group), Each dot shows the value from each independent replicate; **F**–**I** Bio-Plex suspension chip analysis the contents of IL-6, TNF-α, IL-10, and IL-1β (*n* = 8 per group). Each dot shows the value from each independent replicate. Data are the mean ± SEM. Differences were assessed by the Mann–Whitney *U* test. Significance was established at adjusted *P* < 0.05 with a false discovery rate (FDR) of 0.05

It is well known that LPS in the intestinal lumen can be transported across the intestinal epithelial layer into systemic circulation due to increased permeability of the intestinal epithelial barrier. Therefore, we measured the serum levels of lipopolysaccharide. A significant increase was detected in the serum LPS concentration in the HFD-STZ-treated rats compared with vehicle-treated rats, while *P. distasonis* treatment prevented the serum LPS levels from increasing (Fig. 4A). Type 2 diabetes is not only a metabolic disease but also an inflammatory disease [36]. Here, we measured the levels of inflammatory cytokines in rats by qPCR, including TNF-α, IL-1β, and IL-6, which are key proinflammatory factors in type 2 diabetes and closely related to insulin resistance [37]. After HFD and STZ stimulation, qPCR results revealed that the mRNA expression of TNF-α, IL-1β, and IL-6 was significantly decreased in the *P. distasonis*-treated groups compared to the model group, and the mRNA levels of IL-10 were significantly lower than those in the con and PPd groups (Fig. 4B–E). To further verify the transcription levels of inflammatory factors, we used a Bio-Plex suspension chip to quantify the corresponding inflammatory factors in the serum of rats. Consistent with the transcriptomics results, bio-Plex suspension chip analysis revealed that *P. distasonis* suppressed the secretion of TNF-α, IL-1β, and IL-6, and stimulated the secretion of IL-10 (Fig. 4F–I). Collectively, these results demonstrated the anti-inflammatory effect of *P. distasonis*, and the anti-inflammatory ability of the rats in the PPd group was better than that in the TPd group, indicating that the preventive effect of *P. distasonis* was better than the therapeutic effect.

### *P. distasonis* downregulated the TLR4/NF-κB signaling pathway in T2D rats

**Fig. 5:**
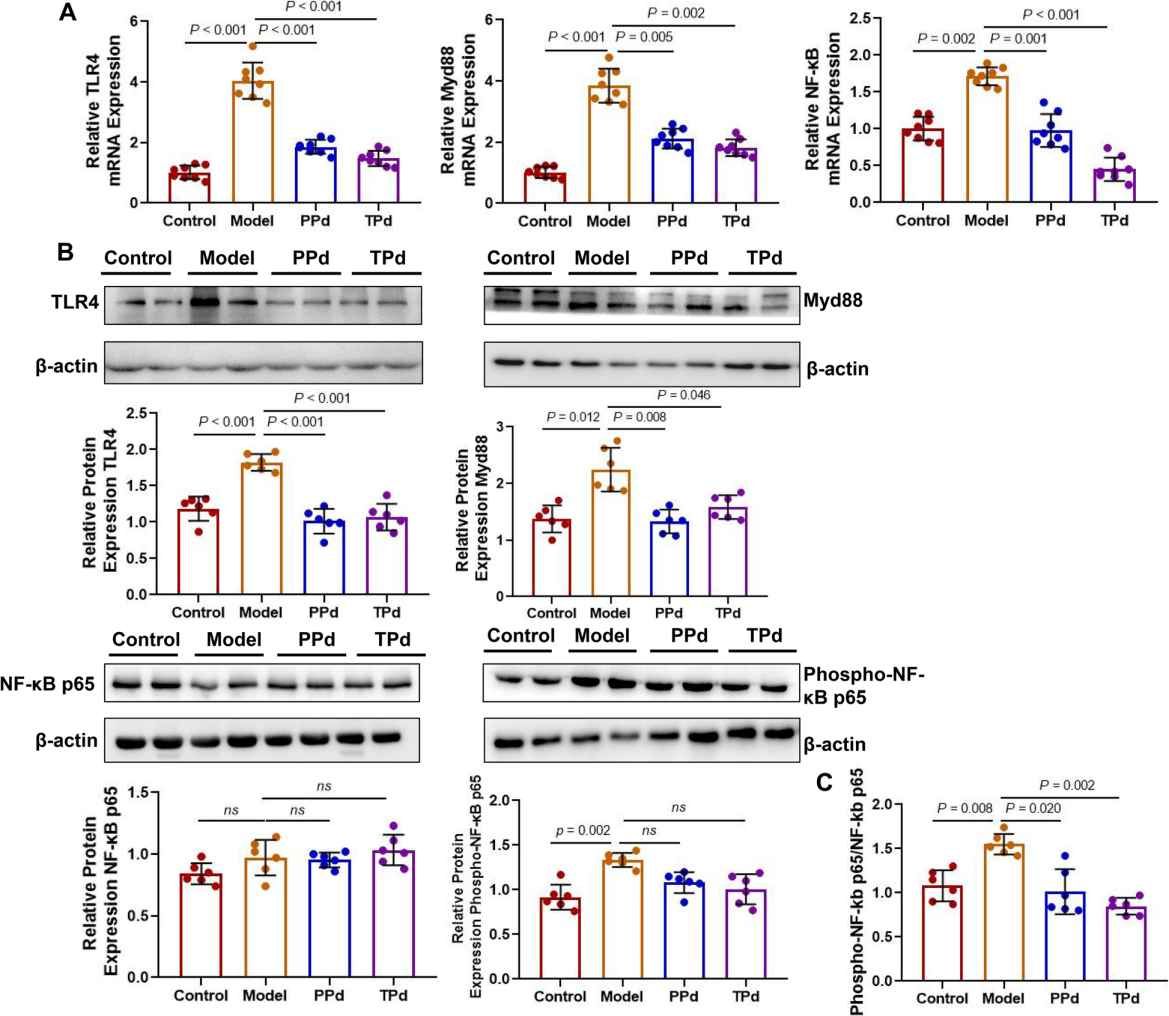
*P. distasonis* inactivates NF-κb signaling via inhibition of TLR4 signaling. **A** Relative mRNA expression of TLR4, Myd88, and NF-κB in colon tissue of rats (*n* = 8 per group). Each dot shows the value from each independent replicate. **B** Representative Western blot and densitometric analyses of TLR4, Myd88, NF-κB pP65, and NF-κB P65 protein in colon tissue of rats (*n* = 6 per group). Each dot shows the value from each independent replicate. **C** The ratio of NF-κB pP65 to NF-κB p65 among all groups (*n* = 6 per group). Data are expressed as mean ± SEM. Differences were assessed by the Mann–Whitney *U* test. Significance was established at adjusted *P* < 0.05 with a false discovery rate (FDR) of 0.05

In recent years, it has been found that the Toll-like receptor 4/Nuclear factor kappa-B (TLR4/NF-κB) pathway is one of the most important pathways in the inflammatory response. TLR4 can specifically recognize LPS and transfer stimulation into cells to activate downstream NF-κB and regulate the synthesis and release of various inflammatory factors [38]. To fully characterize the anti-inflammatory effect of *P. distasonis*, we explored the role of *P. distasonis* in regulating the TLR4-NF-κB signaling pathway. As shown in Fig. 5A, compared with the model group, the transcript level of TLR4, myeloid differentiation factor 88 (Myd88), and NF-κB showed a greater reduction in the PPd and TPd groups. What’s more, the decrease in the TPd group was even more significant than that in the PPd group. As expected, WB results showed that *P. distasonis* intervention significantly alleviated TLR4 and Myd88 increase compared with the model group but had no significant effect on NF-κB P65 and NF-κB pP65 (Fig. 5B). In an attempt to avoid missing any potential effect, the ratio of pP65 to P65 was also used as a measurement of NF-κB activity (Fig. 5C). The results showed that the ratio was lower in the PPd and TPd groups than in the model group. Overall, our data suggested that *P. distasonis* may be involved in the regulation of the TLR4/NF-κB pathway, thereby improving the inflammatory response in T2D rats.

### *P. distasonis* affected tryptophan metabolism in the host

**Fig. 6:**
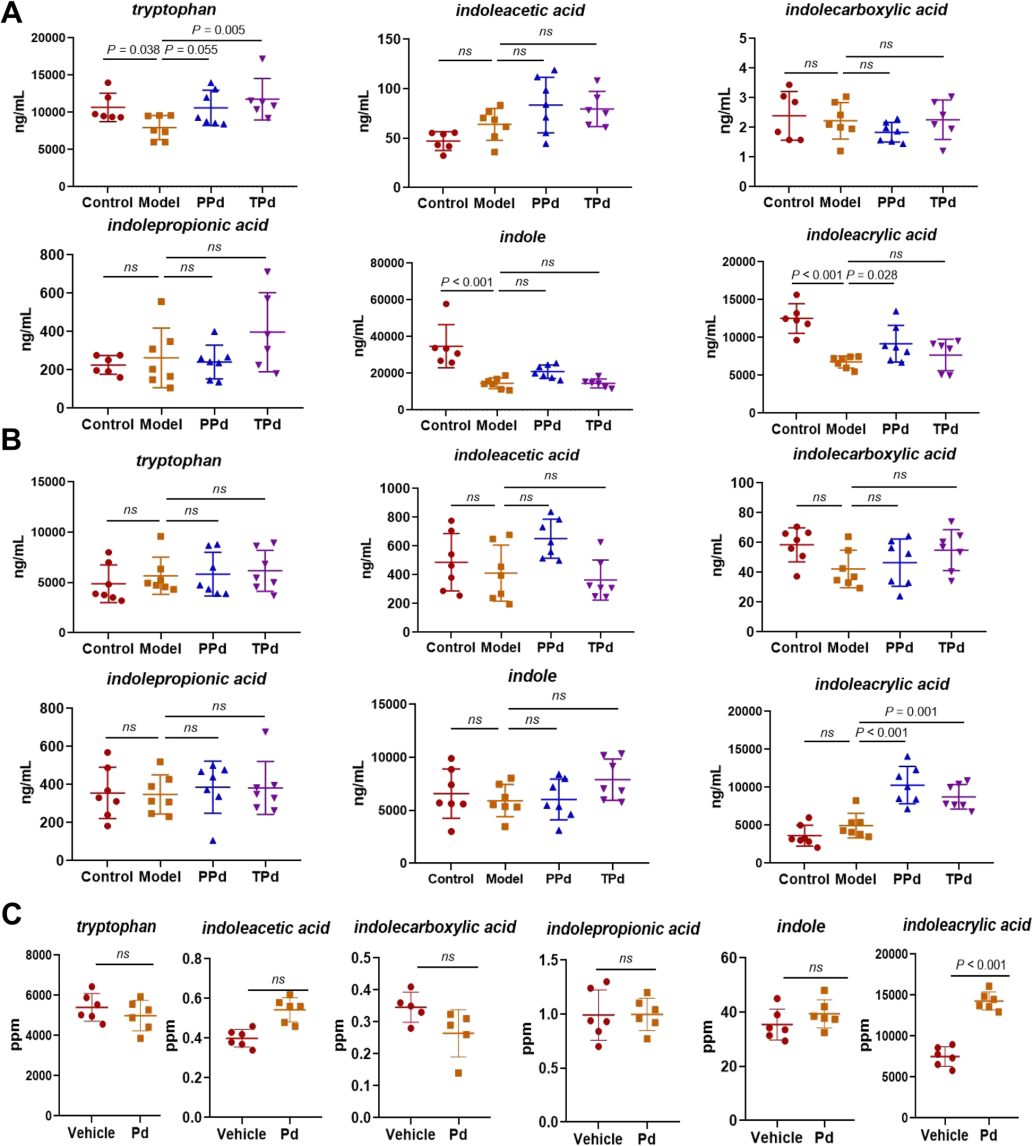
*P. distasonis* modulated the tryptophan metabolism in vivo and in vitro. **A** The levels of tryptophan and indole derivatives in rat serum among all groups (control: *n* = 6, model: *n* = 7, PPd: *n* = 7, TPd: *n* = 6). Each dot shows the value from each independent replicate. **B** The levels of tryptophan and indole derivatives in rat feces among all groups (*n* = 7 per group). Each dot shows the value from each independent replicate. **C** The levels of tryptophan and indole derivatives in the culture extract of *P. distasonis* fermented (*n* = 6 per group). Each dot shows the value from each independent replicate. Data are expressed as mean ± SEM. Differences were assessed by the Mann–Whitney *U* test. Significance was established at adjusted *P* < 0.05 with a false discovery rate (FDR) of 0.05

To explore the mechanism by which *P. distasonis* affects T2D rats, we quantified serum and fecal concentrations of tryptophan and its derivatives. Serum and fecal samples from rats were analyzed using targeted liquid chromatography-tandem mass spectrometry (UPLC-MS) experiments. Compared with the control group, the serum levels of tryptophan, indoleacrylic acid, and indole in the model group were significantly decreased. Whereas, compared with the model group, the serum levels of tryptophan and indoleacrylic acid in the PPd group were significantly increased and the indoleacrylic acid level in the TPd group showed an elevated trend (Fig. 6A). In fecal samples, we found that indoleacrylic acid was only altered in the PPd and TPd rats with a markedly increased level (Fig. 6B). As indicated, administration of *P. distasonis* increased the indoleacrylic acid level in serum and fecal samples. To further explore the mechanism by which *P. distasonis* affects type 2 diabetes, we analyzed the metabolites produced by *P. distasonis.* The culture extract of *P. distasonis* fermented in a BHI medium was determined by UPLC-MS analysis. Compared with the vehicle group, the indoleacrylic acid content was significantly increased in the *P. distasonis* group (Fig. 6C). Based on the above observations, we demonstrated a close association between indoleacrylic acid and *P. distasonis* both in vivo and in vitro.

### *P. distasonis* activated the AhR signaling pathway in vivo

**Fig. 7:**
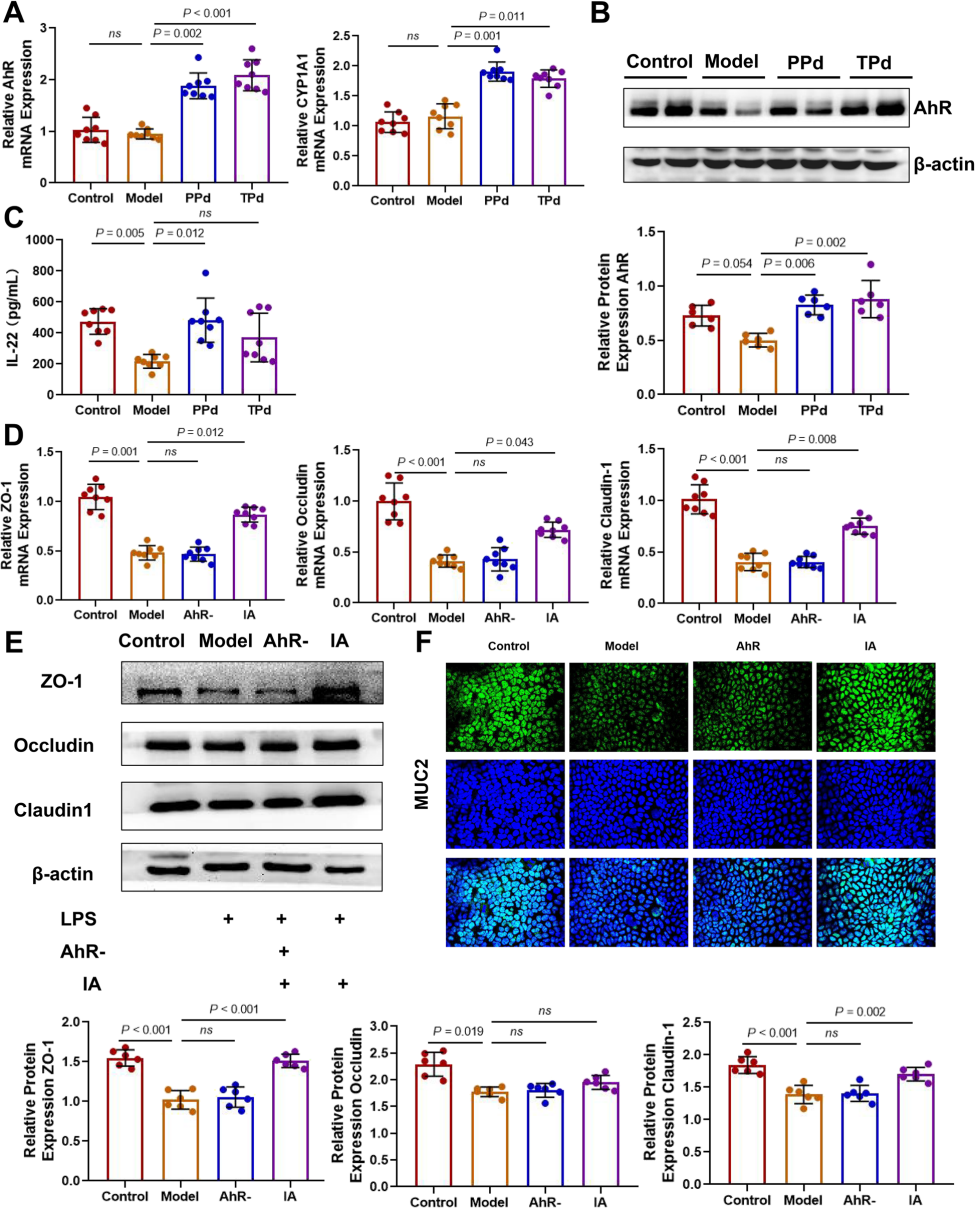
In vitro and in vivo study of the mechanism of *P. distasonis* repairing intestinal barrier. **A** Relative mRNA expression of AhR and its target gene, CYP1A1 in colon tissue of rats (*n* = 8 per group). Each dot shows the value from each independent replicate. **B** Representative Western blot and densitometric analyses of AhR protein in colon tissue of rats (*n* = 6 per group). Each dot shows the value from each independent replicate. **C** The level of IL‑22 in the plasma was measured by ELISA (*n* = 8). Each dot shows the value from each independent replicate. **D** Relative mRNA expression level of Claudin-1, Occludin, and ZO-1 proteins in Caco-2 cells. Each dot shows the value from each independent replicate; **E** Representative Western blot and densitometric analyses of Claudin-1, Occludin, and ZO-1 protein in Caco-2 cells. Each dot shows the value from each independent replicate. **F** Representative pictures of immunofluorescence staining of MUC2 protein in Caco-2 cells (Green is FITC immunofluorescence staining of MUC2, and blue is DAPI staining of nucleus). Data are expressed as mean ± SEM. Differences were assessed by the Mann–Whitney U test. Significance was established at adjusted *P* < 0.05 with a false discovery rate (FDR) of 0.05

According to the literature report [20], indoleacrylic acid is an endogenous AhR ligand. To test whether the alteration of the indoleacrylic acid profile by *P. distasonis* regulates intestinal AhR signaling, we measured the expression levels of AhR and its target gene CYP1A1 in the colon using qPCR. As expected, the levels of AhR and CYP1A1 in *P. distasonis*-treated rats were markedly increased (Fig. 7A). WB analysis of AhR protein expression further confirmed that *P. distasonis* activated the AhR signaling pathway (Fig. 7B). During disease progression, activation of AhR can increase the IL-22 secretion in vivo. IL-22 can sustain the integrity and barrier functions of related tissues such as skin, pancreas, small intestine, colon, liver, lung, and kidney, and prevent damage caused by either invading pathogens or by the inflammatory response itself [39]. Thus, we determined serum IL-22 levels by ELISA. Unsurprisingly, the IL-22 level showed a significant increasing trend in the PPd and TPd groups (Fig. 7C). Taken together, these results supported the concept that the intragastric administration of *P. distasonis* can indeed activate the AhR signaling pathway in rats.

### Indoleacrylic acid enhanced tight junctions in Caco-2 cells

To fully characterize the protective effect of indoleacrylic acid, we explored its role in regulating tight junction proteins in Caco-2 cells after LPS exposure. Related factors were monitored by using WB, qPCR, and immunofluorescence. Following LPS stimulation, RT-PCR revealed that IA treatment increased the expression of Claudin-1, Occludin, and ZO-1 compared to the LPS-stimulated DMSO control, while the effect was counteracted by an aryl hydrocarbon receptor antagonist (Fig. 7D). WB analysis showed that IA incubation significantly prevented the decrease in Claudin-1 and ZO-1 induced by LPS (Fig. 7E) but not in Occludin expression. Similarly, this significant difference disappeared in the antagonist group. Intestinal goblet cells play an important role in the protection of the intestinal epithelium, and MUC2 is closely related to goblet cell function [40]. We wondered whether IA could affect goblet cell function through the AhR signaling pathway. The immunofluorescence results revealed that IA treatment resulted in the upregulation of MUC2 expression, which vanished in the antagonist group (Fig. 7F), further suggesting a link between AhR activation and goblet cell function. In summary, IA alleviated the downregulation of Occludin, Claudin-1, ZO-1, and MUC2 expression induced by LPS at the transcriptome and proteome levels, which showed a positive effect on the intestinal TJ barrier.

## Discussion

With an increasing understanding of the complex interactions between gut microbiota and host, there is an urgent need to identify the specific functions of individual-specific bacteria in disease and health. In this study, we showed that *P. distasonis* was a highly effective commensal bacterium improving T2D in HFD- and STZ-induced rats.

As a metabolic disease, the onset of diabetes is multifactorial. Poor dietary habits are the main influencing factors that directly change the intestinal ecology. Currently, there are very limited studies investigating the relationship between T2D and gut microbiota. Studies have confirmed that dysregulation of the flora structure in T2D leads to abnormal glucose metabolism: poor dietary habits directly cause the imbalance of intestinal flora, and the increased harmful bacteria can damage intestinal mucosa and reduce barrier function [41]. The intestinal barrier relies on the interaction of tight junction proteins (Occludin, Claudin1, ZO-1, etc.) among epithelial cells to limit the entry of toxin pathogens [42]. It plays a key role in preventing the translocation of pathogenic microbial antigens and toxic substances from the intestinal lumen into the systemic circulation [43]. Research confirms that a high-fat diet causes disruption of intestinal barrier functional connexin and occludin, which affects the gut microbiota [44]. Further studies found that the disruption of intestinal barrier permeability was eliminated after antibiotic treatment. *Bifidobacterium* and other polysaccharides play a positive role in protecting intestinal mucosal barrier function and reducing metabolic endotoxin occurrence [45]. Some studies found that *Akkermansia* was significantly increased in T2D patients in fecal flora analysis, and it was speculated that *Akkermansia* may destroy the intestinal mucosal barrier by degrading mucin to produce H_2_S gas and other effects, which could intensify the occurrence of endotoxemia related to intestinal flora and nonspecific inflammatory reactions in the body [46]. This study found that the mechanism by which *P. distasonis* improves T2D is closely related to its function in repairing the intestinal barrier.

LPS, as a component of the cell wall of gram-negative bacteria, is an endotoxin. Using 16 s sequencing, we found that the abundance of gram-negative bacteria such as *Prevotella*, *Acinetobacter*, and *Proteus* increased in T2D rats. Therefore, we speculated that the increase in serum LPS levels in the model group rats might be caused by the imbalance of intestinal flora. Studies have shown that a high-fat diet can promote the LPS level increasing and newly synthesized chylomicrum transportation from intestinal epithelial cells to other tissues, resulting in metabolic endotoxemia [47]. Toll-like receptors are involved in innate immunity by collecting microbiota and hosts. As the natural ligand of LPS, TLR4 plays an important role in regulating LPS metabolism and influencing body weight [48]. LPS is linked to the protein complex CD14/TLR4, which was associated and detected by the innate immune system. It is transported to the recognition receptor Toll-like receptor 4/myeloid differentiation protein 2 (MD-2) complex (TLR4/MD-2) with the help of CD14, which activates TLR-4 and depends on path excitation by MyD88. The active transcription factor NF-κB enters the cytoplasm and binds to DNA, activating the expression of interferon and inflammatory factors such as TNF-α, IL-1β, IL-6, and IL-8, causing a series of nonspecific inflammatory reactions [49–53]. The elevation of LPS depends on TLR to further activate the NF-κB pathway, promoting the release of inflammatory factors, leptin adipose resistin expression, and macrophage M1 proinflammatory phenotype changes, further leading to abnormal insulin resistance and glucose metabolism [54]. Our experimental results proved that *P. distasonis* can inhibit the TLR4-NF-κB signaling pathway and reduce the expression of inflammatory factors in vivo. We then investigated how *P. distasonis* exerts its pharmacological activities.

The specific components of intestinal microflora that improve the intestinal mucosal barrier are not clear. Metabolites derived from the gut microbiota, such as bile acid derivatives, short-chain fatty acids, amino acid derivatives, and liposaccharides, are important signaling molecules linking the gut microbiota with the host [55, 56]. To explore the mechanism of action of *P. distasonis* on T2D, we investigated the metabolites produced by *P. distasonis*. UPLC-MS was utilized to analyze the fermentation broth from *P. distasonis*, revealing that *P. distasonis* can promote the production of IA, a secondary metabolite of tryptophan. Tryptophan, one of the nine so-called essential amino acids, is the only amino acid that contains the structure of indole and can be catabolized by gut bacteria to produce a variety of indole derivatives, which were suggested to enhance intestinal epithelial barrier functions by increasing the expression of genes involved in the maintenance of epithelial cell structure and function [57, 58]. More recent studies suggested that indole-containing molecules strengthen epithelial barrier function via the increased expression of tight junction-related proteins such as claudins and occludins [59]. However, the specific molecular mechanisms of this regulation have yet to be fully elucidated.

Tryptophan metabolites such as indole-3-aldehyde, kynurenine, indole-3-acetic acid, and tryptamine have been shown as ligands for aryl hydrocarbon receptors, and AhR signaling is activated upon the binding [60–63]. Thus, we speculated that the efficacy of IA in modulating TJ barrier integrity and function might depend on the activation of AhR. In addition, microbial tryptophan metabolites signal through AhR to increase IL-22 secretion by innate-like lymphoid cells and T cells, leading to protection from chemically induced colitis. IL-22 signaling leads to increased fucosylation of intestinal mucin [21, 61, 64]. In a model of experimental Citrobacter rodentium infection, Il-22 − / − mice showed severe intestinal epithelial-layer damage, together with systemic bacterial burden and substantially increased mortality [65]. Thus, AhR activation up-regulates IL-22 production, which is crucial for epithelial layer integrity. In this study, we found that IL-22 expression was significantly increased in the PPd group. Interestingly, we also found that IA can directly activate AhR in Caco-2 cells to repair intestinal epithelial function in our present study. Taken together, the protective effect of *P. distasonis* treatment on type 2 diabetes may be in part due to the production of these tryptophan metabolites, potentially leading to the increased differentiation and expression of barrier integrity cell-associated genes such as Claudin-1, Occludin, ZO-1, and MUC2.

Limitation: There is some limitation in this study. First, although our experiment demonstrated that *P. distasonis* has an alleviating effect on type 2 diabetes, several differences were present between the two types of intervention, suggesting that the mechanisms of action of the two regimens may differ, which requires further investigation. Second, the route of administration for *P. distasonis* was oral gavage, and the colonization of the strain was still unknown. Understanding the colonization helps us to explain how bacteria perform their functions. At present, fluorescent probes targeting bacteria can achieve visual analysis of microbiota. The bacterial cell wall is composed of membrane layers and a rigid yet flexible scaffold called peptidoglycan (PG). Hsu et al. designed and introduced a family of novel molecular probes, fluorescent D-amino acids (FDAAs), which were covalently incorporated into PG through the activities of endogenous bacterial transpeptidases [66]. In this way, the colonization of the bacteria in the host could be observed directly, and we will try to use this method to investigate the colonization of *P. distasonis* in the future. Third, our experiments demonstrated that IA could alleviate type 2 diabetes by activating AhR to repair the intestinal barrier in vitro, but in vivo experiments were needed to further verify this conclusion. Therefore, in the next plan, we will focus on the effect of IA and *P. distasonis* on type 2 diabetic rats under the condition of AhR knockout or not.

## Conclusions

In summary, we demonstrated the beneficial effects of the gut commensal *P. distasonis* on T2D. As a chronic inflammatory disease, inflammation in T2D may be caused by harmful substances produced by unbalanced gut microbiota penetrating the intestinal barrier. High-throughput sequencing results revealed that the intestinal tract of T2D rats was enriched with more gram-negative bacteria, such as *Prevotella*, *Acinetobacter*, and *Proteus*. As a cell wall component of gram-negative bacteria, LPS potently activates the NF-κB signaling pathway via TLR4, while *P. distasonis* promotes intestinal barrier repair, decreases blood LPS levels, and inhibits the TLR4-NF-κB signaling pathway. Further researches reported that *P. distasonis* was involved in the tryptophan metabolic pathway. *P. distasonis* could significantly increase the level of indoleacrylic acid (IA) in vivo and in vitro, which activated AhR, thus improving the expression of IL-22 to achieve beneficial effects. Interestingly, IA supplementation significantly enhanced the expression of intestinal barrier-related proteins by activating AhR in Caco-2 cells. In conclusion, this work opens avenues for experimental investigation of gut microbiota in T2D rat model and other metabolic diseases, which provides insights for new preventive or curative treatments for T2D in the clinic.

## Supplementary Information

**Additional file 1: Figure S1. **The Pearson correlation analysis among *Parabacteroides distasonis* and biochemical indicators (*p < 0.05，**p < 0.01，***p< 0.001). Correlation heatmap analysis was applied to assess the association between gut microbiota and T2DM-related indexes. The data of intestinal flora were obtained by high-throughput 16s sequencing, and the data processing method was the same as in this article. The biochemical indexes were obtained by kit, automatic biochemical analyser, and suspension chip method. Pearson’s correlation coefficient was calculated by SPSS (Statistical Product and Service Solutions) statistics 19.0.

**Additional file 2: Figure S2.** The influence of HLJDT intervention on the level of tryptophan and indole derivatives in type 2 diabetic rats (*n* = 7 per group). Each dot shows the value from each independent replicate. Data are expressed as mean ± SEM. Differences were assessed by the Mann-Whitney U test. Significance was established at adjusted *P* < 0.05 with a false discovery rate (FDR) of 0.05. The targeted metabolomics data were obtained by HPLC-MS. All parameters and conditions were the same as those described in this paper. The data were from serum samples collected from rats at week 10.

**Additional file 3: Table S1 **Sequences of amplification primers (rats).

**Additional file 4: Table S2 **Sequences of amplification primers (Caco-2 cell).

**Additional file 5: Table S3 **The information of antibodies.

**Additional file 6: Table S4 **Retention times, diagnostic MRM transitions and optimized instrument settings (Retention Time, RT; Collision Energy, CE; Dwell Time, DT).

**Additional file 7: Table S5 **Biochemical indexes of renal function and lipid level.

**Additional file 8: Figure S3.** AOD changes of ZO-1 and Occludin expression in the colon of rats at week 12 (n = 6 per group). Data are expressed as mean ± SEM. Differences were assessed by the Mann-Whitney U test. Significance was established at adjusted *P* < 0.05 with a false discovery rate (FDR) of 0.05. AOD: Average optical density.

**Additional file 9: **Animal model and study design of Figure S1 and S2.docx.

**Additional file 10: **Original WB images.pptx.

## Data Availability

All data generated or analyzed during this study are included in this article or its supplementary information files. 16 s sequencing data are also publicly available through the SRA database, accession number PRJNA942238[67].

## Abbreviations

LPS: Lipopolysaccharide
T2D: Type 2 diabetes
IDs: Indole derivatives
ILCs: Innate lymphoid cells
HFD: High-fat diet
STZ: Streptozotocin
IA: Indoleacrylic acid
AhR: Aryl hydrocarbon receptor
CFU: Colonyformingunit
PBS: Phosphate buffered saline
OGTT: Oral glucose tolerance test
FBG: Fasting blood glucose
DMEM: Dulbecco’s modified Eagle’s medium
RIPA: Radioimmunoprecipitation assay
MRM: Multiple reaction monitoring
OTUs: Operational taxonomic units
LEfSe: Linear discriminant analysis effect size
GSP: Glycated serum protein
ALB: Albumin
BUN: Blood urea nitrogen
PCoA: Principal co-ordinates analysis
LDA: Linear discriminant analysis
TJ: Tight junction
TLR4: Toll-like receptor 4
NF-κB: Nuclear factor kappa-B
Myd88: Myeloid differentiation fritter 88
TG: Total cholesterol
TC: Triglyceride
LDL-C: Low-density lipoprotein cholesterol
HDL-C: High-density lipoprotein cholesterol
