# Supplementary material for: Indoleacrylic acid produced by Parabacteroides distasonis alleviates type 2 diabetes via activation of AhR to repair intestinal barrier
Source: BMC Biol. 2023 Apr 18;21:90. doi: 10.1186/s12915-023-01578-2 (PMC10114473; doi:10.1186/s12915-023-01578-2)
Supplement: Supplementary file 1 — Additional file 1: Figure S1. The Pearson correlation analysis among Parabacteroides distasonis and biochemical indicators (*p < 0.05，**p < 0.01，***p< 0.001). Correlation heatmap analysis was applied to assess the association between gut microbiota and T2DM-related indexes. The data of intestinal flora were obtained by high-throughput 16s sequencing, and the data processing method was the same as in this article. The biochemical indexes were obtained by kit, automatic biochemical analyser, and suspension chip method. Pearson’s correlation coefficient was calculated by SPSS (Statistical Product and Service Solutions) statistics 19.0. [file 12915_2023_1578_MOESM1_ESM.docx]

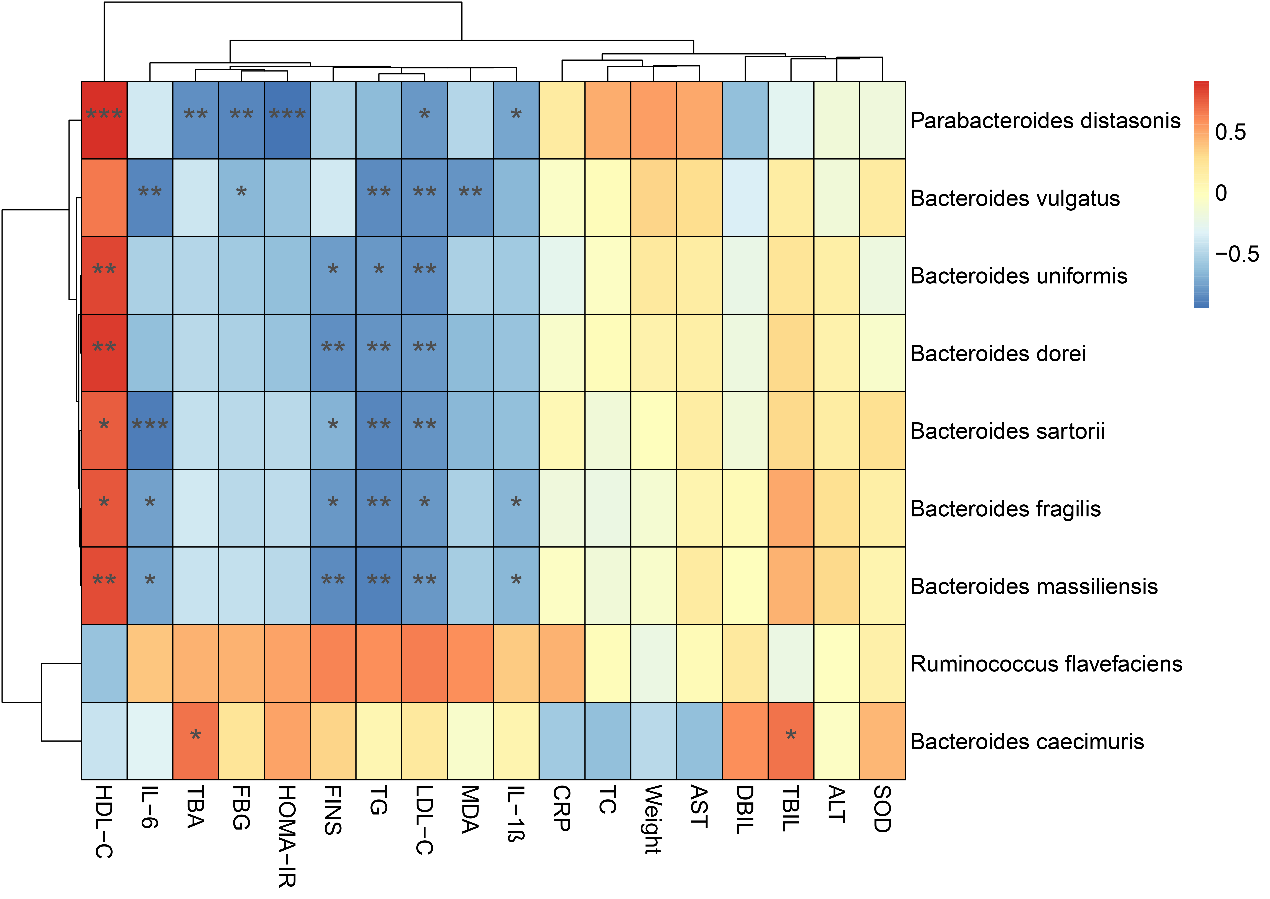


Figure S1. The Pearson correlation analysis among *Parabacteroides distasonis* and biochemical indicators (*p < 0.05，**p < 0.01，***p < 0.001). Correlation heatmap analysis was applied to assess the association between gut microbiota and T2DM-related indexes. The data of intestinal flora were obtained by high-throughput 16s sequencing, and the data processing method was the same as in this article. The biochemical indexes were obtained by kit, automatic biochemical analyser and suspension chip method. Pearson’s correlation coefficient was calculated by SPSS (Statistical Product and Service Solutions) statistics 19.0.
