# Supplementary material for: Indoleacrylic acid produced by Parabacteroides distasonis alleviates type 2 diabetes via activation of AhR to repair intestinal barrier
Source: BMC Biol. 2023 Apr 18;21:90. doi: 10.1186/s12915-023-01578-2 (PMC10114473; doi:10.1186/s12915-023-01578-2)
Supplement: Supplementary file 2 — Additional file 2: Figure S2. The influence of HLJDT intervention on the level of tryptophan and indole derivatives in type 2 diabetic rats (n = 7 per group). Each dot shows the value from each independent replicate. Data are expressed as mean ± SEM. Differences were assessed by the Mann-Whitney U test. Significance was established at adjusted P < 0.05 with a false discovery rate (FDR) of 0.05. The targeted metabolomics data were obtained by HPLC-MS. All parameters and conditions were the same as those described in this paper. The data were from serum samples collected from rats at week 10. [file 12915_2023_1578_MOESM2_ESM.docx]

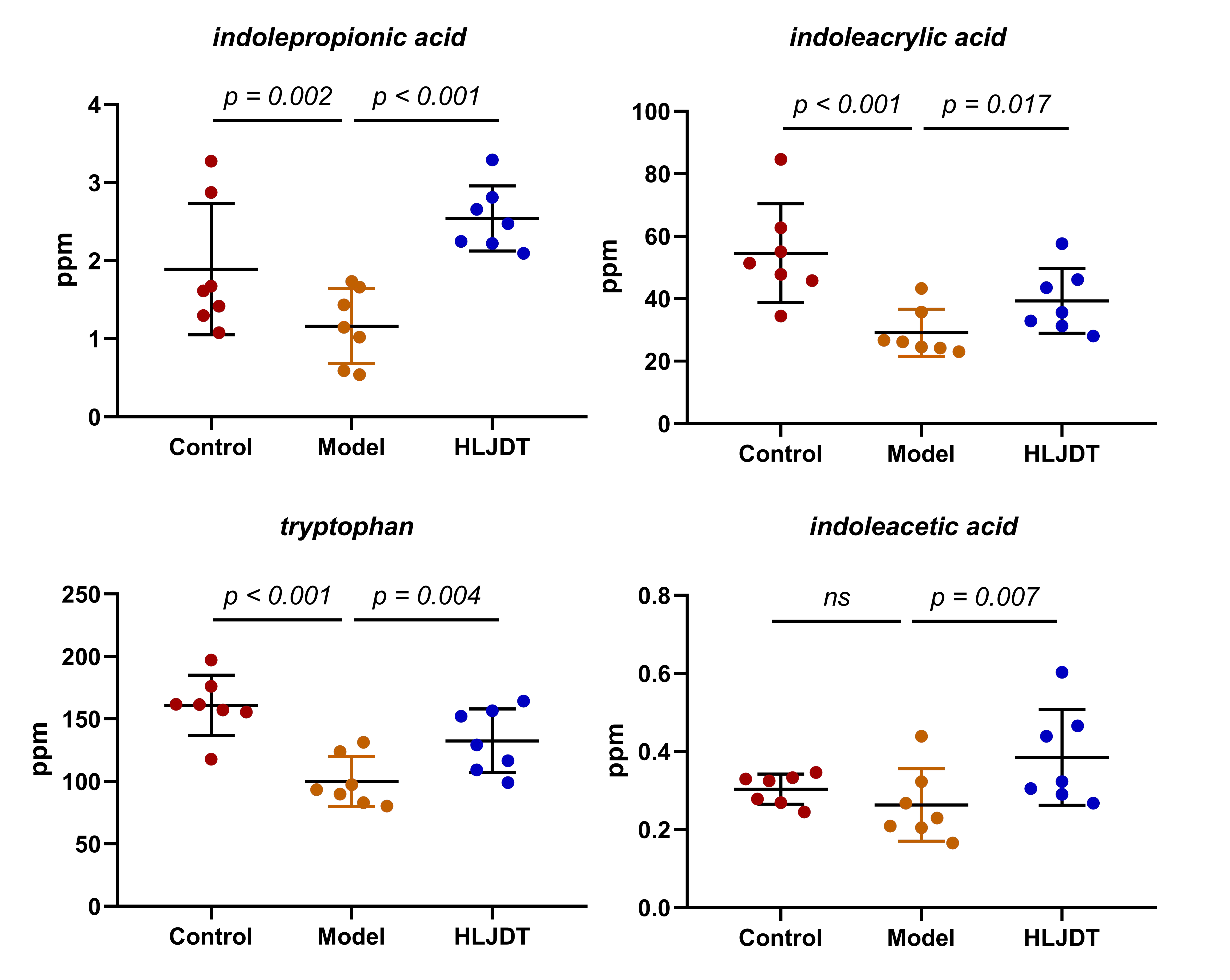


Figure S2. The influence of HLJDT intervention on the level of tryptophan and indole derivatives in type 2 diabetic rats (n = 7 per group). Each dot shows the value from each independent replicate. Data are expressed as mean ± SEM. Differences were assessed by the Mann-Whitney U test. Significance was established at adjusted P < 0.05 with a false discovery rate (FDR) of 0.05. The targeted metabolomics data were obtained by HPLC-MS. All parameters and conditions were the same as those described in this paper. The data were from serum samples collected from rats at week 10.
