# Supplementary material for: Indoleacrylic acid produced by Parabacteroides distasonis alleviates type 2 diabetes via activation of AhR to repair intestinal barrier
Source: BMC Biol. 2023 Apr 18;21:90. doi: 10.1186/s12915-023-01578-2 (PMC10114473; doi:10.1186/s12915-023-01578-2)
Supplement: Supplementary file 3 — Additional file 3: Table S1 Sequences of amplification primers (rats). [file 12915_2023_1578_MOESM3_ESM.docx]

**Table S1** Sequences of amplification primers (rats)

| Gene | direction | Primer (5’→3’) |
| --- | --- | --- |
| GAPDH | Forward | TGGCAAAGTGGAGATTGTTGCC |
|  | Reverse | AAGATGGTGATGGGCTTCCCG |
| ZO-1 | Forward | CCACCTCGCACGTATCACAAGC |
|  | Reverse | GGCAATGACACTCCTTCGTCTCTG |
| Claudin-1 | Forward | GGACAACATCGTGACTGCTCAGG |
|  | Reverse | TGCCAATTACCATCAAGGCTCTGG |
| Occludin | Forward | TCGTGATGTGCATCGCTGTATTCG |
|  | Reverse | CGTAACCGTAGCCGTAACCGTAAC |
| Claudin-2 | Forward | GCAAACAGGCTCCGAAGATACT |
|  | Reverse | GAGATGATGCCCAAGTACAGAG |
| TNF-α | Forward | CACCGGCAAGGATTCCAA |
|  | Reverse | CACTCAGGCATCGACATTCG |
| IL-6 | Forward | TTCCAGCCAGTTGCCTTCTTG |
|  | Reverse | ATCCTCTGTGAAGTCTCCTCTCC |
| IL-10 | Forward | AGGCAGTGGAGCAGGTGAAG |
|  | Reverse | ACGTAGGCTTCTATGCAGTTGATG |
| IL-1β | Forward | GGATGATGACGACCTGCTAGTG |
|  | Reverse | CACTTGTTGGCTTATGTTCTGTCC |
| TLR4 | Forward | TATAAAACATAAGGAGAGGAGG |
|  | Reverse | ATCCGAGATGTGGAACTGGC |
| Myd88 | Forward | ACCGCATCGAGGAGGACTG |
|  | Reverse | CTGTGGGACACTGCTCTCCA |
| NF-κB | Forward | GATTGAGATGATTTTGGAG |
|  | Reverse | GTATGTTAAGTATATGATTG |
| AhR | Forward | GCCAGGACCAGTGTAGAGC |
|  | Reverse | ATTCAGCGCCTGTAACAAGAA |
| CYP1A1 | Forward | GACCCTTACAAGTATTTGGTCGT |
|  | Reverse | GGTATCCAGAGCCAGTAACCT |
