# Supplementary material for: Indoleacrylic acid produced by Parabacteroides distasonis alleviates type 2 diabetes via activation of AhR to repair intestinal barrier
Source: BMC Biol. 2023 Apr 18;21:90. doi: 10.1186/s12915-023-01578-2 (PMC10114473; doi:10.1186/s12915-023-01578-2)
Supplement: Supplementary file 4 — Additional file 4: Table S2 Sequences of amplification primers (Caco-2 cell). [file 12915_2023_1578_MOESM4_ESM.docx]

**Table S2** Sequences of amplification primers (Caco-2 cell)

| Gene | direction | Primer (5’→3’) |
| --- | --- | --- |
| β-actin | Forward | AAAGACCTGTACGCCAACACAGTGCTGTCTGG |
|  | Reverse | CGTCATACTCCTGCTTGCT GATCCACATCTGC |
| ZO-1 | Forward | ATCCCTCAAGGAGCCATTC |
|  | Reverse | CACTTGTTTTGCCAGGTTTTA |
| Claudin-1 | Forward | AAGTGCTTGGAAGACGATGA |
|  | Reverse | CTTGGTGTTGGGTAAGAGGTT |
| Occludin | Forward | CCAATGTCGAGGAGTGGG |
|  | Reverse | CGCTGCTGTAACGAGGCT |
