# Supplementary material for: Indoleacrylic acid produced by Parabacteroides distasonis alleviates type 2 diabetes via activation of AhR to repair intestinal barrier
Source: BMC Biol. 2023 Apr 18;21:90. doi: 10.1186/s12915-023-01578-2 (PMC10114473; doi:10.1186/s12915-023-01578-2)
Supplement: Supplementary file 5 — Additional file 5: Table S3 The information of antibodies. [file 12915_2023_1578_MOESM5_ESM.docx]

**Table S3** The information of antibodies

| **Antibodies** | **Source** | **Catalogue code** | **Dilutions** | **Manufacturer** |
| --- | --- | --- | --- | --- |
| Occludin | Rabbit | DF7504 | 1:1000 (WB) | Affinity Biosciences |
| Claudin-1 | Rabbit | ab15098 | 1:1000 (WB) 1:200 (IHC-P) | abcam |
| Claudin-2 | Rabbit | ab125293 | 1:1000 (WB) | abcam |
| ZO-1 | Rabbit | ab221547 | 1:1000 (WB) 1:500 (IHC-P) | abcam |
| TLR4 | Rat | ab95562 | 1:1000 (WB) | abcam |
| Myd88 | Rabbit | AF5195 | 1:1000 (WB) | Affinity Biosciences |
| NF-κB P65 | Rabbit | AF5006 | 1:1000 (WB) | abcam |
| NF-κB pP65 | Rabbit | AF2006 | 1:1000 (WB) | abcam |
| AhR | Rabbit | A1451 | 1:1000 (WB) | ABclonal |
| MUC2 | Rabbit | DF8390 | 1:1000 (WB) 1:200 (IF) | Affinity Biosciences |
| beta Actin | Rabbit | ab8227 | 1:1000 (WB) | abcam |
| anti-rabbit | Goat | S0001 | 1:1000 (WB) 1:200 (IHC-P) | Affinity Biosciences |
| anti-rat | Goat | S0009 | 1:1000 (WB) | Affinity Biosciences |
| Alexa Fluor 488-labeled anti-rabbit | Goat | A0423 | 1:200 (IF) | Beyotime |
