# Supplementary material for: Indoleacrylic acid produced by Parabacteroides distasonis alleviates type 2 diabetes via activation of AhR to repair intestinal barrier
Source: BMC Biol. 2023 Apr 18;21:90. doi: 10.1186/s12915-023-01578-2 (PMC10114473; doi:10.1186/s12915-023-01578-2)
Supplement: Supplementary file 6 — Additional file 6: Table S4 Retention times, diagnostic MRM transitions and optimized instrument settings (Retention Time, RT; Collision Energy, CE; Dwell Time, DT). [file 12915_2023_1578_MOESM6_ESM.docx]

**Table S4** Retention times, diagnostic MRM transitions and optimized instrument settings (Retention Time, RT; Collision Energy, CE; Dwell Time, DT)

| Analytes | RT (min) | ESI mode | Precursor ion > product ion | CE (eV) | DT (msec) |
| --- | --- | --- | --- | --- | --- |
| tryptophan | 3.79 | + | 205.25>143.05 | -32 | 0.493 |
| indolecarboxylic acid | 3.912 | - | 160.10>40.15 | 41 | 0.493 |
| indoleacetic acid | 3.58 | + | 176.15>116.15 | 35 | 0.493 |
| indolepropionic acid | 4.984 | + | 190.10>116.25 | 40 | 0.493 |
| indoleacrylic acid | 5.12 | + | 188.05>170.15 | -13 | 0.493 |
| Indole | 5.024 | + | 118.10>65.1 | -33 | 0.493 |
| 5-Hydroxyindole-2-carboxylic acid (IS) | 6.083 | - | 176.05>115.20 | 38 | 0.493 |
