# Supplementary material for: Indoleacrylic acid produced by Parabacteroides distasonis alleviates type 2 diabetes via activation of AhR to repair intestinal barrier
Source: BMC Biol. 2023 Apr 18;21:90. doi: 10.1186/s12915-023-01578-2 (PMC10114473; doi:10.1186/s12915-023-01578-2)
Supplement: Supplementary file 7 — Additional file 7: Table S5 Biochemical indexes of renal function and lipid level. [file 12915_2023_1578_MOESM7_ESM.docx]

**Table S5** Biochemical indexes of renal function and lipid level

| Indicators | Control | Model | PPd | TPd |
| --- | --- | --- | --- | --- |
| ALB (g/L) | 30.27±0.98** | 25.38±0.91 | 27.33±0.82**^##^ | 26.15±0.94^##^ |
| BUN (mmol/L) | 6.43±0.54** | 18.10±2.72 | 11.43±2.84**^#^ | 20.08±4.74^##^ |
| TG (mmol/L) | 0.72±0.20** | 6.07±6.89 | 1.11±0.36* | 1.77±0.88* |
| TC (mmol/L) | 1.34±0.22* | 3.52±3.0 | 1.08±0.24* | 1.70±0.70* |
| LDL-C (mmol/L) | 0.25±0.03* | 0.79±0.69 | 0.21±0.06* | 0.34±0.19* |
| HDL-C (mmol/L) | 0.67±0.08 | 0.53±0.24 | 0.59±0.10 | 0.52±0.211 |

ALB: Albumin; BUN: Blood urea nitrogen; TG: Total cholesterol; TC: Triglyceride; LDL-C: low density lipoprotein cholesterol; HDL-C: High density lipoprotein cholesterol. (compared with the model group：*p < 0.05，**p < 0.01; compared with the control group：#p < 0.05，##p < 0.01).
