# Supplementary material for: Indoleacrylic acid produced by Parabacteroides distasonis alleviates type 2 diabetes via activation of AhR to repair intestinal barrier
Source: BMC Biol. 2023 Apr 18;21:90. doi: 10.1186/s12915-023-01578-2 (PMC10114473; doi:10.1186/s12915-023-01578-2)
Supplement: Supplementary file 8 — Additional file 8: Figure S3. AOD changes of ZO-1 and Occludin expression in the colon of rats at week 12 (n = 6 per group). Data are expressed as mean ± SEM. Differences were assessed by the Mann-Whitney U test. Significance was established at adjusted P < 0.05 with a false discovery rate (FDR) of 0.05. AOD: Average optical density. [file 12915_2023_1578_MOESM8_ESM.docx]

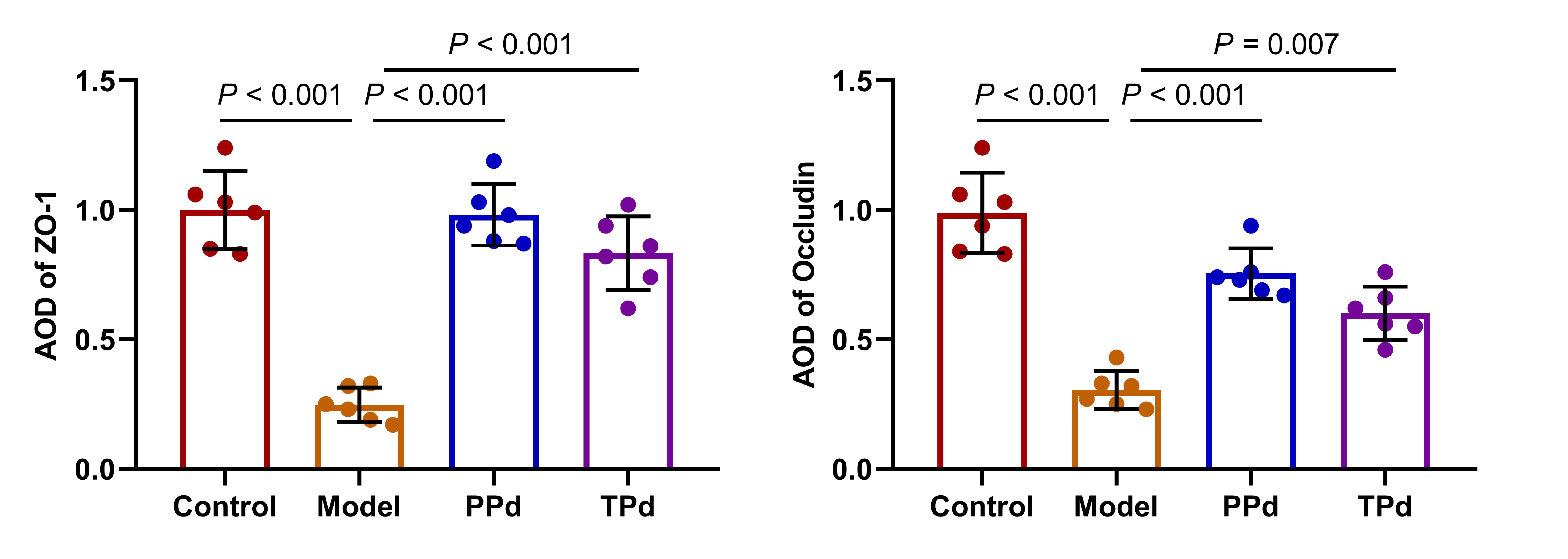


Figure S3. AOD changes of ZO-1 and Occludin expression in the colon of rats at week 12 (n = 6 per group). Data are expressed as mean ± SEM. Differences were assessed by the Mann-Whitney U test. Significance was established at adjusted P < 0.05 with a false discovery rate (FDR) of 0.05. AOD: Average optical density.
