# Supplementary material for: Indoleacrylic acid produced by Parabacteroides distasonis alleviates type 2 diabetes via activation of AhR to repair intestinal barrier
Source: BMC Biol. 2023 Apr 18;21:90. doi: 10.1186/s12915-023-01578-2 (PMC10114473; doi:10.1186/s12915-023-01578-2)
Supplement: Supplementary file 9 — Additional file 9: Animal model and study design of Figure S1 and S2.docx. [file 12915_2023_1578_MOESM9_ESM.docx]

Animal model and study design of Figure S1 and S2

A total of 32 rats were randomly divided into control group (control, n = 8), model group (model, n = 12) and treatment group (HLJDT, n = 12). The model group and treatment group fed with high-fat diet for 4 weeks and received a single intraperitoneal injection of STZ. Two weeks after the injection of STZ, rats in the treatment group were given HLJDT extract for 4 weeks. The control and model groups were treated with corresponding volumes of saline i.p.
