# Supplementary material for: Indoleacrylic acid produced by Parabacteroides distasonis alleviates type 2 diabetes via activation of AhR to repair intestinal barrier
Source: BMC Biol. 2023 Apr 18;21:90. doi: 10.1186/s12915-023-01578-2 (PMC10114473; doi:10.1186/s12915-023-01578-2)
Supplement: Supplementary file 10 — Additional file 10: Original WB images.pptx. [file 12915_2023_1578_MOESM10_ESM.pptx]

## Slide 1
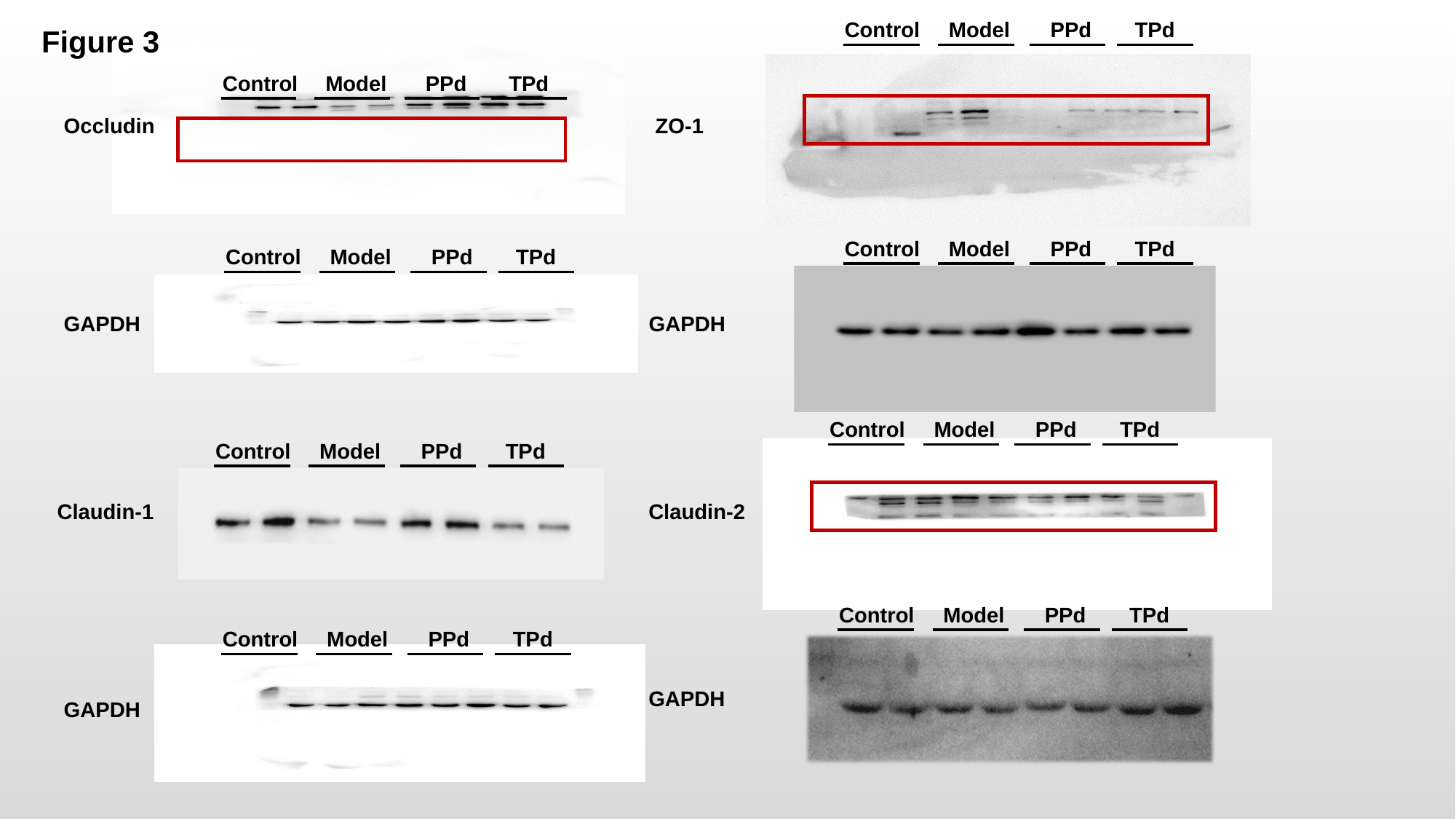

Control
Model
PPd
TPd
Figure 3
Control
Model
PPd
TPd
Occludin
ZO-1
Control
Model
PPd
TPd
Control
Model
PPd
TPd
GAPDH
GAPDH
Control
Model
PPd
TPd
Control
Model
PPd
TPd
Claudin-2
GAPDH
Claudin-1
GAPDH
Control
Model
PPd
TPd
Control
Model
PPd
TPd

## Slide 2
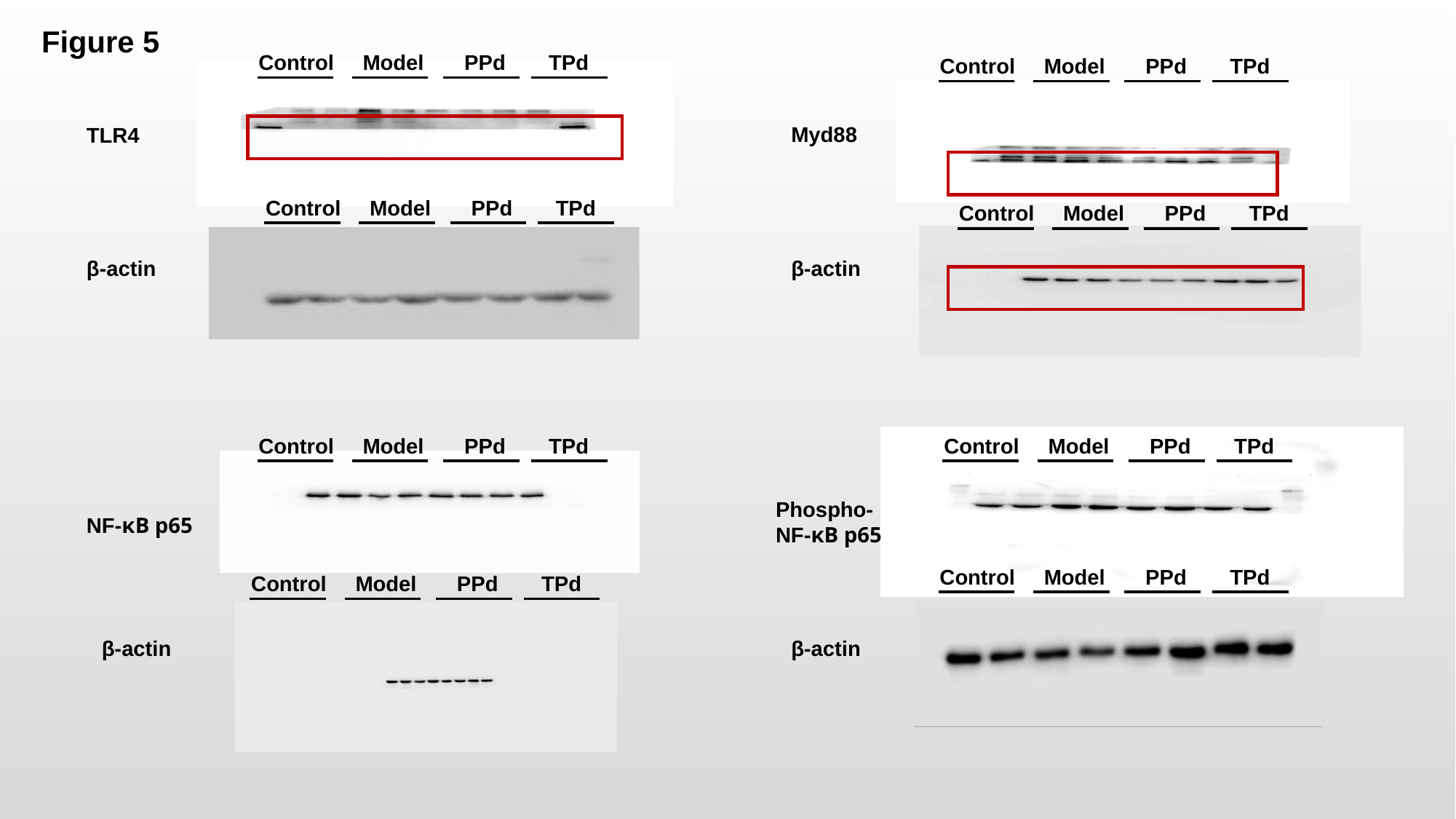

Figure 5
Control
Model
PPd
TPd
Control
Model
PPd
TPd
Myd88
TLR4
Control
Model
PPd
TPd
Control
Model
PPd
TPd
β-actin
β-actin
Control
Model
PPd
TPd
Control
Model
PPd
TPd
Phospho-NF-κB p65
NF-κB p65
Control
Model
PPd
TPd
Control
Model
PPd
TPd
β-actin
β-actin

## Slide 3
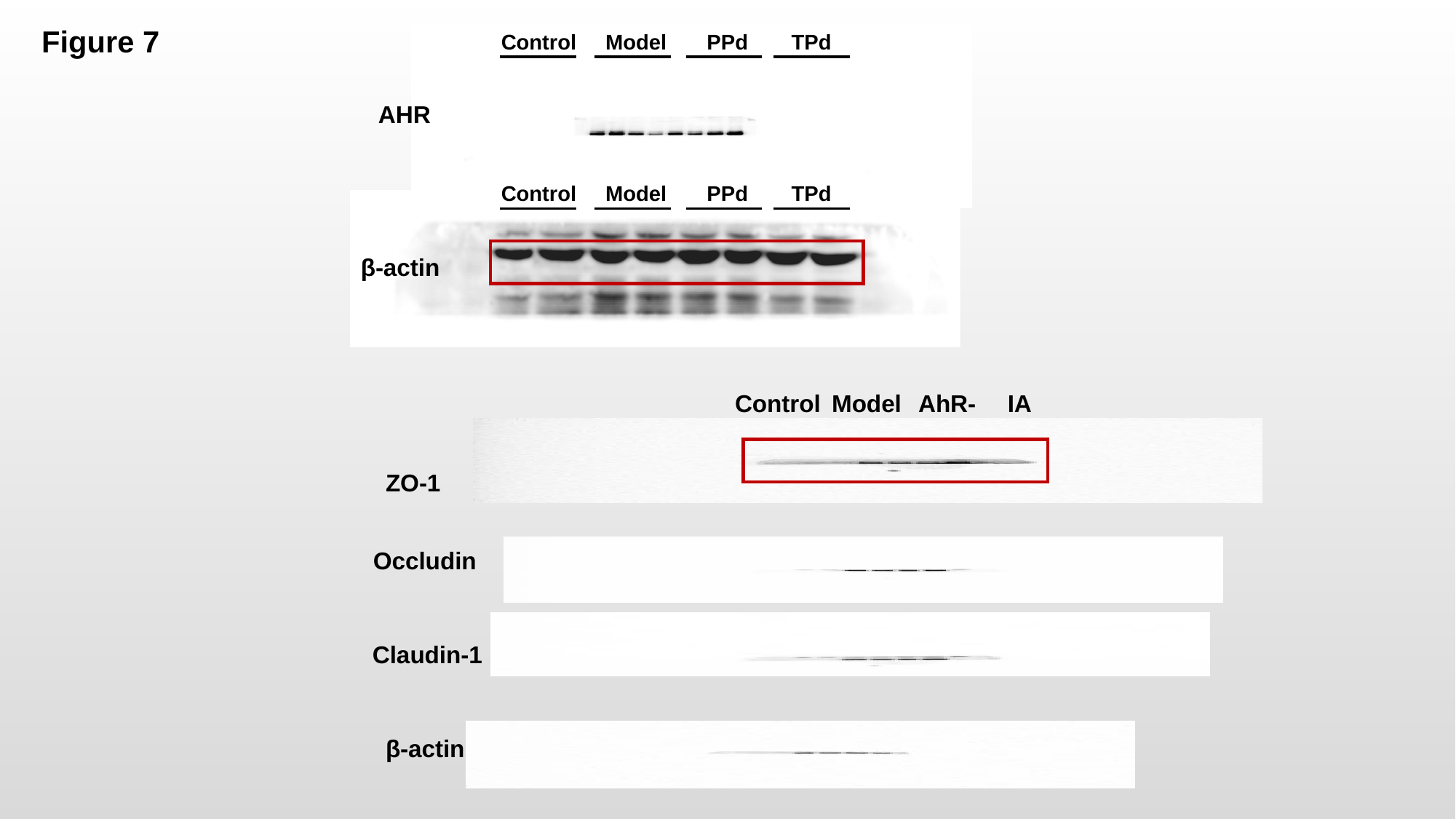

Figure 7
Control
Model
PPd
TPd
AHR
Control
Model
PPd
TPd
β-actin
Control
Model
AhR-
IA
ZO-1
Occludin
Claudin-1
β-actin
